# Nematode-Trapping Fungi Produce Diverse Metabolites during Predator–Prey Interaction

**DOI:** 10.3390/metabo10030117

**Published:** 2020-03-20

**Authors:** Ting-Hao Kuo, Ching-Ting Yang, Hsin-Yuan Chang, Yen-Ping Hsueh, Cheng-Chih Hsu

**Affiliations:** 1 Department of Chemistry, National Taiwan University, Taipei 10617, Taiwan; 2 Institute of Molecular Biology, Academia Sinica, Taipei 115, Taiwan

**Keywords:** nematode-trapping fungi, *Arthrobotrys*, *Caenorhabditis elegans*, predator–prey interaction, metabolomics, molecular networking, *Arthrobotrys musiformis* trap-associated peptide, desferriferrichrome, linoleyl alcohol, nonadecanamide, citicoline

## Abstract

Nematode-trapping fungi are natural antagonists of nematodes. These predatory fungi are capable of switching their lifestyle from a saprophytic to predatory stage in the presence of nematodes by developing specialized trapping devices to capture and consume nematodes. The biochemical mechanisms of such predator–prey interaction have become increasingly studied given the potential application of nematode-trapping fungi as biocontrol agents, but the involved fungal metabolites remain underexplored. Here, we report a comprehensive liquid–chromatography mass spectrometry (LC–MS) metabolomics study on one hundred wild isolates of nematode-trapping fungi in three different species, *Arthrobotrys* *oligospora, Arthrobotrys thaumasia*, and *Arthrobotrys musiformis*. Molecular networking analysis revealed that the fungi were capable of producing thousands of metabolites, and such chemical diversity of metabolites was notably increased as the fungi switched lifestyle to the predatory stage. Structural annotations by tandem mass spectrometry revealed that those fungal metabolites belonged to various structural families, such as peptide, siderophore, fatty alcohol, and fatty acid amide, and their production exhibited species specificity. Several small peptides (<1.5 kDa) produced by *A.* *musiformis* in the predatory stage were found, with their partial amino acid sequences resolved by the tandem mass spectra. Four fungal metabolites (desferriferrichrome, linoleyl alcohol, nonadecanamide, and citicoline) that were significantly enriched in the predatory stage were identified and validated by chemical standards, and their bioactivities against nematode prey were assessed. The availability of the metabolomics datasets will facilitate comparative studies on the metabolites of nematode-trapping fungi in the future.

## 1. Introduction

Nematode-trapping fungi (NTF) are an intriguing class of carnivorous microorganisms that are capable of developing specialized predatory devices to trap and digest nematodes [1]. Importantly, the predatory behavior not only acts as a response to a nutrient-deprived condition but also promotes the cycling of biomass in a food web. More than 200 species of NTF have been described to date, and they are broadly distributed in terrestrial and aquatic ecosystems [2]. NTF are also natural antagonists of nematodes, which are crucial crop pests and cause agricultural damage worldwide. The global financial loss due to plant-parasitic nematodes is estimated to be over USD 100 billion [3,4]. To address this problem, NTF, therefore, serve as ecophysiological sources of novel biological control agents that are beneficial to agriculture and ecosystems. 

The predatory behavior adapted by NTF is intriguing. Initiated by a chemical eavesdropping of the nematode pheromones, the fungus can sense pheromones of nematodes, switch from saprophytic to predacious lifestyles, and create three-dimensional trapping devices to ensnare the nematodes [5]. The trapping devices have diverse morphological forms. For example, *Arthrobotrys oligospora*, one of the best-studied NTF, creates adhesive networks to immobilize and then digest nematodes [6]. The underlying physiochemical processes in developing the trapping devices are of great interest in biological research. Studies on genomic, transcriptomic, and proteomic aspects have collectively shown that the trap formation is associated with the upregulation of multiple signal transduction pathways, biosynthesis of adhesive proteins [7], intercellular communication [8], and nitrate assimilation [9]. On the other hand, little is known about the metabolome of NTF. Previous studies have shown that *A. oligospora* produces volatile organic compounds (VOCs) that mimic sex and food cues to attract the nematode prey in the absence of nematodes [10], and a more recent study also reported VOCs produced by *A. oligospora* during the predation process [11]. Nonetheless, the metabolites produced by NTF induced by the nematode prey during predation remained largely unexplored.

**Figure 1:**
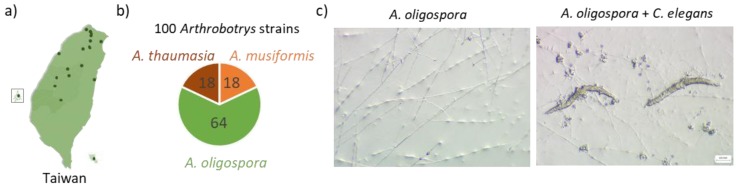
A large-scale collection of 100 strains of nematode-trapping fungi of *Arthrobotrys* genus in Taiwan. (**a**) The locations of which the nematode-trapping fungi were isolated in Taiwan. (**b**) The collection of 100 strains of nematode-trapping fungi of *Arthrobotrys* genus, including 18 strains of *A. thaumasia*, 18 strains of *A. musiformis*, and 64 strains of *A. oligospora*. (**c**) The nematode-trapping fungi in the saprophytic stage (left) and in the predatory stage (right).

To gain more insights into how NTF response to nematode prey during predation, we analyzed the metabolites of 100 wild isolates of three different NTF species, *Arthrobotrys oligospora*, *Arthrobotrys thaumasia*, and *Arthrobotrys musiformis,* which were all collected in Taiwan [12] (Figure 1a,b; Table S1). All of these 100 wild isolates are capable of forming traps to capture the nematode prey, and interestingly, they also exhibited significant polymorphic predatory ability [12]. These *Arthrobotrys* fungi change the lifestyle from the saprophytic stage to the predatory stage, forming trapping devices to capture and digest the nematodes [13] (Figure 1c). In this study, we aim to unravel specialized metabolites produced by those NTF, particularly in the predatory stage via a liquid–chromatography tandem mass spectrometry (LC–MS/MS) untargeted metabolomics approach. We utilized the Global Natural Product Social Molecular Networking (GNPS) [14] to facilitate data processing. Fungal metabolites that were potentially accountable for ingesting nematodes were rigorously validated, and the biological functions of these candidate metabolites were tested with the model nematode *Caenorhabditis elegans.*

## 2. Results

### 2.1. Revealing Chemical Diversity of Metabolites of Nematode-Trapping Fungi in Different Life Stages With Molecular Networking

**Figure 2:**
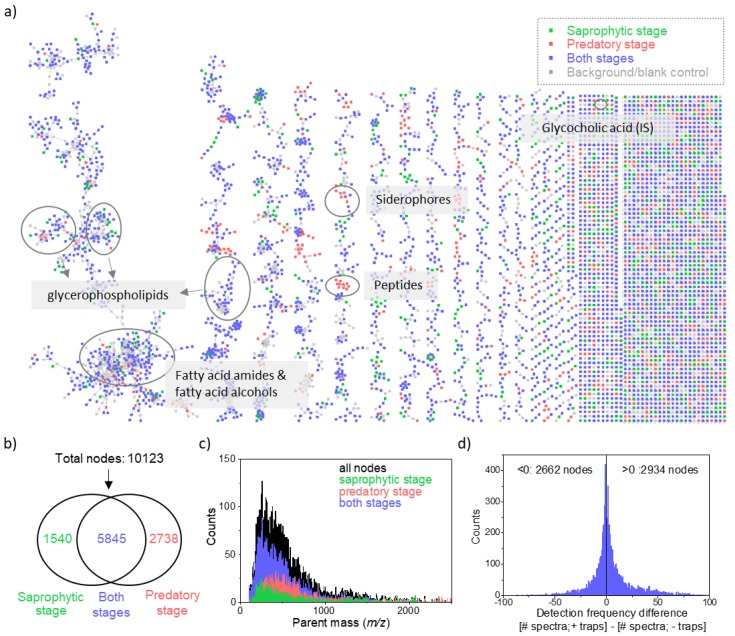
*Arthrobotrys* molecular network: visualization of metabolic diversity during the fungi-nematode interaction. (**a**) The molecular network of the isolated 100 *Arthrobotrys* strains (organic extract). The putatively identified metabolites are marked. Node colors represent sample groups at which the metabolites were detected. Nodes are colored based on whether they were found in the saprophytic stage (green), predatory stage (red), or in both stages (blue). Grey nodes originate from blank controls of media, worms, and internal standard (glycocholic acid). The molecular network of the aqueous extract is shown in Figure S1. (**b**) The Venn diagram and (**c**) *m*/*z* distribution of the parent mass of the 10,123 nodes in the molecular networks in Figure 2a and Figure S1. Nodes in the blank control samples were excluded. (**d**) The difference in the detection frequency of the blue nodes between the predatory stage (+traps) and the saprophytic stage (−traps). Nodes with positive *x*-axis values represented that they were more frequently present in the predatory stage.

To obtain metabolomic signatures of NTF in different life stages, we cultured 100 *Arthrobotrys* fungal strains in the presence or absence of *C. elegans*, representing the saprophytic and predatory life stages, respectively. We sequentially extracted the cultures by organic and aqueous solvent and examined the metabolites by untargeted LC–MS/MS. The resulting metabolomics dataset, which encompassed 470,343 MS/MS spectra, was processed through the GNPS molecular networking online infrastructure. As a result, the detected fungal metabolites were visualized as molecular networks basing on their tandem mass spectral similarities (Figure 2a for the organic extract; Figure S1 for the aqueous extract). In a molecular network, metabolites are represented by nodes (i.e., each node represents a consensus MS/MS spectrum of a metabolite), and connected if having similar MS/MS spectra above the threshold cosine value. In this way, metabolites with the same structural moieties tend to cluster and form a molecular family in the network. This strategy is beneficial for annotating metabolites as well as discovering novel structural analogues, and has been widely applied in examining specialized metabolites from plants [15] and microbes [16]. Here we showed that the *Arthrobotrys* metabolomic dataset yielded 12,179 nodes and hundreds of molecular clusters spanning in the molecular network (Figure 2a and Figure S1). Node colors highlighted the metabolites from different life stage(s). In conclusion, we found 10,123 nodes in the fungal extracts, not in blank controls (Figure 2b), and these nodes possessed a large range of molecular weights (Figure 2c). Among the nodes, up to 58% were consistently existing across the two life stages (blue nodes), 15% were detected in the saprophytic stage (green nodes), and 27% were uniquely detected when the fungi had formed the traps in the predatory stage (red nodes). The results indicated that NTF were capable of producing a large variety of metabolites, and their diversity was greatly increased due to lifestyle switching that induced the formation of the trapping devices.

### 2.2. Annotation of Arthrobotrys musiformis Trap-Associated Peptides (AmTPs)

**Figure 3:**
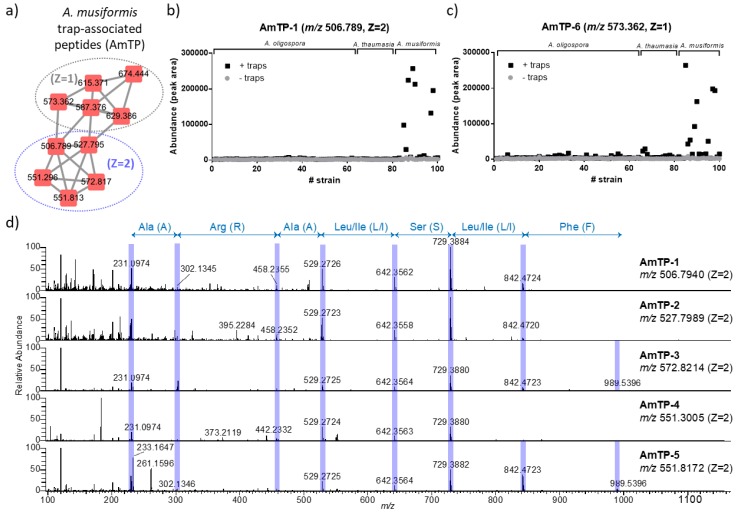
The annotation of *Arthrobotrys musiformis* trap-associated peptides (AmTPs). (**a**) The molecular cluster of the *Arthrobotrys musiformis* trap-associated peptides (AmTPs). The parent ion *m*/*z* and charge stage (Z) were shown. (**b**,**c**) The abundance of the AmTPs in each *Arthrobotrys* strain before and after forming the traps (exemplified by a doubly charged AmTP-1 in (**b**); a singly charged AmTP-6 in (**c**)). The peptides were specific to the strains of the species *A. musimorfis*. Abundance was estimated by the LC–MS peak area. (**d**) Tandem mass spectrometric interrogation of the doubly-charged *A. musiformis* peptides. The fragments indicating amino acid sequences are highlighted.

We first focused on the metabolites uniquely detected in the predatory stage as these metabolites are most likely to be critical for the biochemical functions of the trapping devices. To this end, we investigated the chemical identities of thousands of red nodes in the network. In fact, there were a number of molecular clusters merely made up of red nodes (predatory stage only), but their molecular identities remain unknown by searching against the GNPS database. Then we thoroughly inspected their tandem mass spectra in an attempt to speculate their identities. Eventually, a peptide cluster was assigned (Figure 3a; highlighted in Figure 2a) because the metabolites possess representative MS/MS fragments indicative of the molecular weights of amino acid residues. Notably, these novel small peptides (with molecular weights <1.5 kDa) were uniquely detected in *A. musiformis* in the predatory stage, but not in *A. oligospora* nor *A. thaumasia* (Figure 3b,c), and we thus annotated these peptides as *A. musiformis* trap-associated peptide (AmTP). Interestingly, the production levels of a given peptide showed substantial variation among different *A. musiformis* strains, implying that the production of NTF metabolites is not only species-specific but also strain-specific. Likewise, in our recent study, we also observed that prey-sensing induced trap morphogenesis is a highly polymorphic trait in *A. oligospora* and *A. musiformis* [12]. In this peptide cluster, we further resolved two structural subfamilies: doubly-charged peptides and singly-charged peptides. Additionally, we found the conserved amino acid sequences in each peptide subfamily. The doubly-charged peptides possessed a common amino acid sequence of Ala-Arg-Ala-Leu/Ile-Ser-Leu/Ile (Figure 3d), and we denoted these five peptides as AmTP-1 to AmTP-5;meanwhile, the five singly-charged peptides had another common sequence of Leu/Ile-Ser-Leu/Ile (Figure S2), and were denoted as AmTP-6 to AmTP-10. There has not been a report showing that *Arthrobotrys* NTF are capable of producing small peptides. To realize whether the AmTPs resembled any known peptide, we searched their amino acid sequences against the public peptide databases, including the Antimicrobial Peptide Database (APD), PeptideAtlas, and PepBank, but none of the amino acid sequences was found aligned with reported peptides. Finally, the possible full amino acid sequences of the 10 AmTPs and the structural forms (linear or cyclic), which were putatively annotated based on the high-resolution MS/MS spectra, are supplemented in Table S2 and require validation with purified compounds or synthetic chemical standards. To sum up, the presence of AmTPs implied that NTF exhibited species-specific metabolites that might contribute to the predatory activities of different fungal species.

### 2.3. Identifying Fungal Metabolites Significantly Enriched in the Predatory Stage

We next investigated the blue nodes (metabolites detected in both saprophytic and predatory stages) and focused on those compounds showing an enrichment in the predatory stage. We identified that over 50% of the blue nodes (2934 out of 5845 nodes) were detected more frequently in the fungi in the predatory stage than in the saprophytic stage (Figure 2d). Through quantitative analysis, those nodes with significant enrichment in the predatory stage were sorted out and annotated by GNPS spectral libraries. The putative structural identities were validated by chemical standards and finally reported herein. To sum up, we successfully identified four metabolites that were significantly enriched in the predatory stage: desferriferrichrome, linoleyl alcohol, nonadecanamide, and citicoline (Tables S3 and S4). Their locations in the molecular network, mass spectrometric characteristics, structural analogues, and bioactivities were elaborated in detail in the following.

#### 2.3.1. Desferriferrichrome

**Figure 4:**
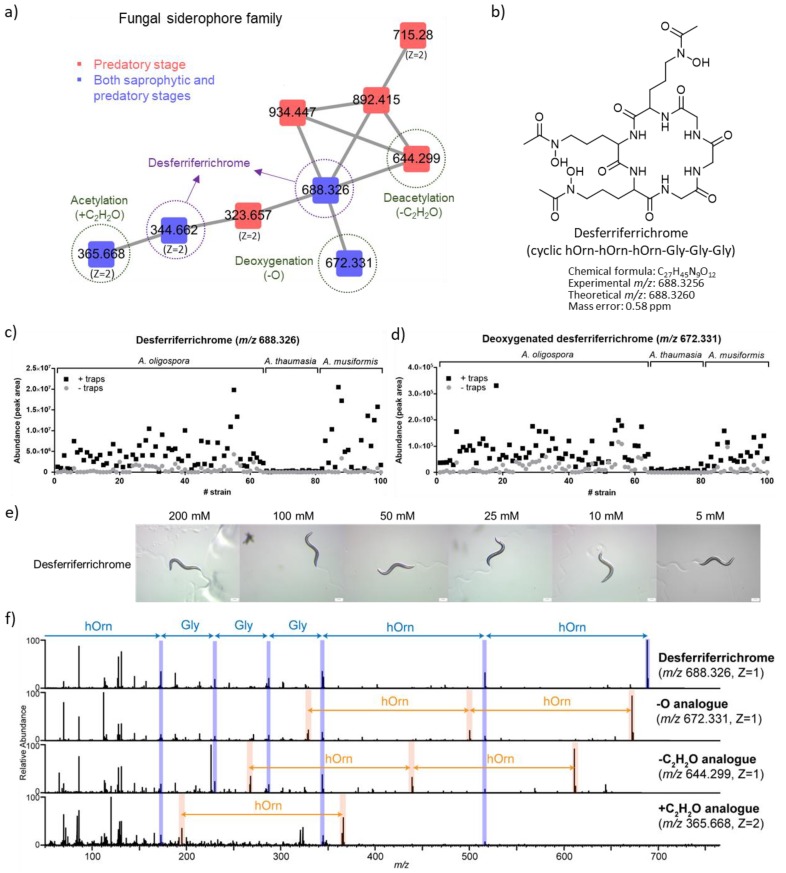
The fungal siderophore (desferriferrichrome) and its structural analogues. (**a**) The molecular family of desferriferrichrome. The nodes representing desferriferrichrome and three structural analogues were highlighted. The *m*/*z* values of the nodes are shown, and the doubly-charged nodes are particularly denoted as Z = 2. (**b**) The chemical structure of desferriferrichrome: a cyclic peptide composed of three glycine (Gly) monomers and three modified ornithine residues with hydroxamate groups (hOrn). (**c**,**d**) The abundance of the siderophores (exemplified by desferriferrichrome in (**c**); deoxygenated analogue in (**d**)) in each *Arthrobotrys* strain before and after forming the traps was shown. The level of the siderophores was decreased in the *A. thaumasia* strains. Abundance was estimated by the LC–MS peak area. (**e**) The nematicidal activity test of desferriferrichrome. (**f**) Tandem mass spectrometric interrogation of desferriferrichrome and its structural analogues. The fragments indicating hydroxamating ornithine or glycine monomer are highlighted.

In a small cluster in the molecular network (Figure 4a; highlighted in Figure 2a), a blue node with *m*/*z* 688.326 (Z = 1, [M+H]^+^, 687.318 Da) was identified by the GNPS database as desferriferrichrome, a reported fungal siderophore [17,18,19,20]. This identification was further validated by high-resolution LC–MS with the chemical standard (Figure S3). Desferriferrichrome is a hydroxamate siderophore composed of three modified ornithine residues with hydroxamate groups (hOrn) and three glycine (Gly) monomers (Figure 4b). Interestingly, desferriferrichrome was preferentially produced by two species, *A. oligospora* and *A. musiformis*, but not *A. thaumasia* (Figure 4c). This suggests that the regulation of the production of desferriferrichrome is species-specific. We further tested whether desferriferrichrome exhibited any biological activities in *C. elegans* and found that this compound does not inhibit the growth of the nematodes, suggesting that it has little toxicity towards the nematode prey (Figure 4e).

In the same molecular cluster, we further annotated three nodes with *m*/*z* 644.299 (Z = 1, [M+H]^+^, 643.291 Da), *m*/*z* 672.331 (Z = 1, [M+H]^+^, 671.323 Da), and *m*/*z* 365.668 (Z = 2, [M+2H]^2+^, 729.328 Da) as structural analogues of desferriferrichrome based on the characteristic fragments indicative to hOrn and Gly monomers in the MS/MS spectra (Figure 4f and Figure S3e). Based on the elemental compositions predicted by high-resolution *m*/*z* values, the three nodes were further identified as the deoxygenated (−O), acetylated (+C_2_H_2_O), and deacetylated (−C_2_H_2_O) analogues of desferriferrichrome, respectively (Table S3). Moreover, the three analogues of desferriferrichrome were also preferentially produced by *A. oligospora* and *A. musiformis*, whereas two of the analogues (the deoxygenated and deacetylated analogues) were enriched in the predatory stage, consistent with desferriferrichrome (Figure 4d and Figure S3d). It has been reported that the mechanism of iron releasing and uptaking with desferriferrichrome involved its acetylated analogue [21], while the roles of the deoxygenated and deacetylated analogues remain unexplored.

#### 2.3.2. Linoleyl Alcohol

**Figure 5:**
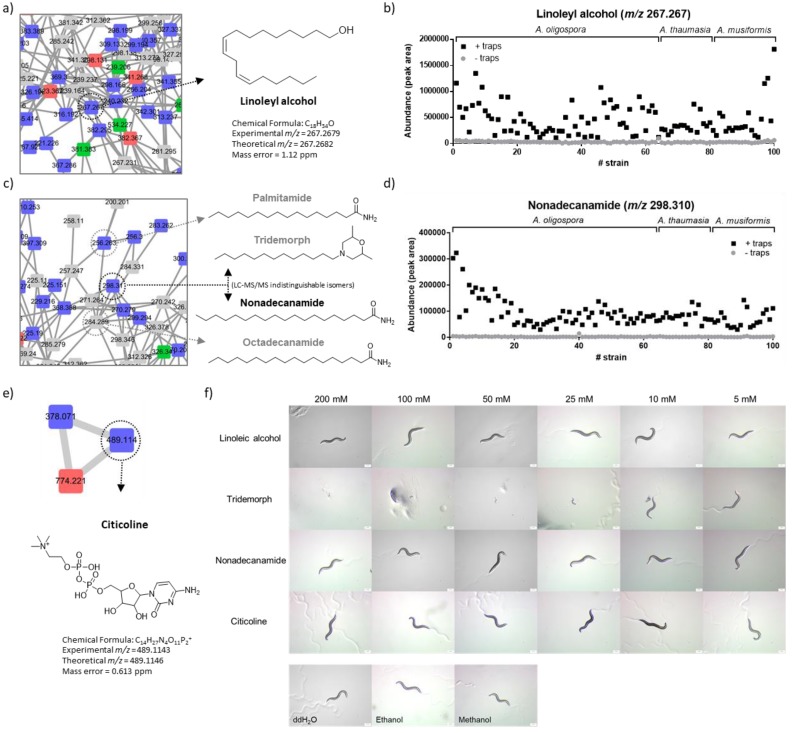
The identified fungal metabolites enriched in the predatory stage. Three nodes enriched in the predatory stage were identified and tested for their nematicidal activity. Linoleyl alcohol: (**a**) Highlighted molecular network with linoleyl alcohol. (**b**) The abundance of linoleyl alcohol in each *Arthrobotrys* strain before and after the formation of the traps. Nonadecanamide: (**c**) Highlighted molecular network with the node with *m*/*z* 298.310, which was identified as nonadecanamide. Tridemorph, a structural isomer of nonadecanamide with the identical LC–MS/MS information, was also shown. (**d**) The abundance of nonadecanamide in each *Arthrobotrys* strain before and after the formation of the traps. Citicoline: (**e**) The molecular cluster with citicoline (from the aqueous extract). (**f**) The nematicidal activity test of the identified metabolites. Negative control experiments using pure solvent were shown. Only tridemorph showed significant inhibition activity against nematodes.

In the largest molecular cluster that contained plenty of fatty acid amides and fatty acid alcohols (putatively assigned by the GNPS database), a blue node with *m*/*z* 267.267 was identified as linoleyl alcohol, a polyunsaturated C18:2 fatty alcohol (Figure 5a). The identification was also validated by the chemical standard (Figure S4). Interestingly, its abundance was low in the fungal cultures without the traps but drastically enriched with the formation of the traps in almost all of the strains (Figure 5b). However, this metabolite showed no significant nematicidal activity (Figure 5f).

#### 2.3.3. Nonadecanamide

In the same molecular cluster, another node with *m*/*z* 298.310 was initially annotated by GNPS as a morpholine derivative named tridemorph (Figure 5c). This metabolite was enriched after the formation of traps for most of the NTF strains (Figure 5d). To validate this annotation, we showed that the LC–MS measurement of the chemical standard of tridemorph was consistent with the node with *m*/*z* 298.310 (Figure S5). Although we found tridemorph had significant nematicidal activity to the worms (Figure 5f), the identification seemed to be suspicious because tridemorph is a synthetic pesticide invented by BASF in the 1960s (under the trade name of Calixin) [22]. Whether this compound originated from the fungus was thus questioned. In this regard, we postulated that *m*/*z* 298.310 might be a structural isomer of tridemorph. Tracking the node back to the molecular network, we found this metabolite clustered with many fatty acid amides putatively annotated by GNPS (Figure 5a). Based on the chemical formula (C_19_H_39_NO), we proposed an alternative identity of the compound as nonadecanamide, a saturated fatty acid primary amide with a linear C19:0 fatty acyl chain (see Figure 5c). This annotation was further supported by mass spectrometric validation with the chemical standard of nonadecanamide (Figure S5), whereas nonadecanamide showed no significant nematicidal activity (Figure 5f). 

#### 2.3.4. Citicoline

We identified a node with *m*/*z* 498.114 as citicoline, also known as cytidine diphosphate–choline (CDP–choline) in the molecular network of the aqueous extracts (Figure 5e). This identification was validated by the chemical standard (Figure S6). This compound was enriched after the formation of the traps in many for the NTF strains (Figure S7). In the molecular network, citicoline was clustered with two nodes, but they were eventually annotated as the in-source fragment/adduct; therefore, no structural analogue was found.

## 3. Discussion

NTF hold great potential as biocontrol agents to target parasitic nematodes, and, thus, are a subject of interest to scientists. The interactions between NTF and their nematode prey have been studied for several decades, but the underlying molecular mechanisms only began to be revealed in the more recent years [5,9,10,11,13]. In this study, we have further expanded our understanding of the metabolites of a hundred strains of *Arthrobotrys* NTF by metabolomic analyses. In conclusion, the chemical diversity of the fungal metabolites drastically increases when NTF switch their lifestyle from the saprophytic stage into the predatory stage. Specifically, NTF in response to the lifestyle switch enrich the production of thousands of metabolites, containing hundreds of structural families, as revealed by molecular networking. Our results and previous studies have collectively shown that the lifestyle switching of NTF is accompanied by physiological changes in transcriptome [7,8], proteome [7], and metabolome. Furthermore, such changes in the metabolome exhibit species-specificity. The structurally diverse metabolites enriched in the predatory stage are potentially related to the complicated predatory mechanisms of NTF.

To characterize these enriched metabolites, we have searched the compounds of interest against the GNPS spectral libraries and validated the annotations with chemical standards. As a result, we have successfully characterized the fungal metabolites out of several structural families, which have been reported to be involved in essential biological functions in the other organisms. These metabolites, or their structural families, include citicoline, fatty acid amide class, fatty alcohol class, playing roles in the biosynthesis of phosphatidylcholine (PC) of cell membranes [23], signal transduction [24,25], and synthesis of wax esters or ether glycerolipids [26,27], respectively. The three identified metabolites, linoleyl alcohol, nonadecanamide, and citicoline, might thus enable similar functions in NTF and serve as the indicators of the underlying metabolomic pathways. In this regard, we reason that when NTF switch lifestyle to the predatory stage, the expression of cell signaling molecules and biosynthesis of cell membranes are upregulated, consequently leading to the enrichment of the metabolites. This speculation is also consistent with the previously proposed model for the formation of nematode-trapping devices based on the genomic and proteomic analyses of *A. oligospora* [7].

All the collected strains of *Arthrobotrys* spp. increased the production of linoleyl alcohol in the predatory stage. Nonetheless, the biological function of linoleyl alcohol in fungi is still poorly understood. Our results suggested that linoleyl alcohol has limited inhibition activity towards *C. elegans*. Previous research considered linoleyl alcohol as a substrate for producing linoleic acid through *in vivo* oxidation in the rat brain [28]. Interestingly, linoleic acid was one of the earliest isolated nematicidal compounds of *Arthrobotrys* NTF, and the amount of linoleic acid was positively correlated with the number of trapping devices formed by the NTF in the predatory stage [29]. Yet the LC–MS setup used in this study (compounds were detected in positive ionization mode) required additional chemical derivatization of linoleic acid for its quantification. Collectively, we propose that the observed phenotype in which NTF produced a higher amount of linoleyl alcohol in the predatory stage might be related to the production of linoleic acid as a nematicide to kill the prey via the oxidation of linoleyl alcohol.

Siderophores are iron-chelating compounds that are secreted by microorganisms to uptake irons from the environment. Importantly, siderophores have been related with the pathogenic activity of microbes and, in some cases, they show nematicidal activities against nematodes [30,31,32]. Several putative biosynthetic gene clusters (BGCs) of nonribosomal peptides (NRPs) have been identified in the genome of *A. oligospora*, and one of the BGCs was proposed to involve in siderophore production [7]. Herein, we revealed that both *A. oligospora* and *A. musiformis* were capable of producing desferriferrichrome, a reported hydroxamate siderophore [17,18,19], and its production was significantly increased in the predatory stage. We also annotated an analogous biosynthetic gene cluster (BGC) of desferriferrichrome in the genome of *A. oligospora* TWF154 [12]. The biological activity of desferriferrichrome has not been widely studied [33], whereas we showed that it was ineffective in inhibiting the growth of *C. elegans* in an agar-based assay. 

NTF have been reported to produce various metabolites as nematicidal weapons. To realize whether the identified metabolites enriched in the predatory stage (i.e., linoleyl alcohol, nonadecanamide, citicoline, and desferriferrichrome) were nematicides, we accessed their bioactivities to the larvae of *C. elegans*, which showed no obvious inhibition activity. Further efforts are required in mining the potential nematicidal compounds from the thousands of the uncharacterized NTF metabolites.

Metabolomic studies of NTF are still in the infancy, and, therefore, the number of NTF metabolites encompassed in the current metabolomic databases is somewhat lower. For example, we searched the Natural Products Atlas [34], one of the largest public databases of microbial natural products, and found only three metabolites originated from *Arthrobotrys* fungi. This limitation makes the identification of NTF metabolites very much challenging. To facilitate future studies, our NTF molecular networks have been contributed to GNPS and made accessible to the public. As GNPS enables continuous re-analysis of living data, the unknown metabolites of NTF will be increasingly annotated as more metabolomic datasets are uploaded to GNPS in the foreseeable future. On the other hand, we sought to sort out the uncharacterized metabolites that particularly existed in the predatory stage and were detected in over half of the strains of each NTF species; their putative chemical identities are provided in the supporting information (Tables S5–S8 and Figures S8 and S9, Appendix A, and Appendix B). The chemical information of these uncharacterized but important metabolites, including MS/MS spectral lists, molecular formula predicted by SIRIUS 4.0 [35], and common biological sample sources linking to public metabolomics datasets by MASST [36], may serve as the future reference for NTF research. 

This study provides insights into the metabolomics of NTF upon lifestyle switching. The molecular networking analyses of the 100 *Arthrobotrys* NTF strains have shown that the chemical diversity in metabolites significantly elevates when NTF switch their lifestyle from the saprophytic stage into the predatory stage. Within the thousands of metabolites, we have identified some of the critical ones that are potentially participating in the predatory behaviors of NTF. This study will shed light on biochemical mechanisms of forming trapping devices by NTF and on molecular crosstalk of the predator–prey interaction.

## 4. Materials and Methods

### 4.1. Materials, Reagents, and Chemical Standards

The nematode-trapping fungi were collected from soil samples in Taiwan [12]. The *Caenorhabditis elegans* wild-type strain N2 was purchased from the Caenorhabditis Genetics Center (CGC, University of Minnesota, MN). Acetonitrile was purchased from J. T. Baker (Phillipsburg, NJ, USA). Ethyl acetate was purchased from Macron Fine Chemicals (Center Valley, PA, USA). Formic acid was purchased from Honeywell Fluka (Morris Plains, NJ, USA), and methanol was purchased from Duksan Pure Chemicals (Seonggok-dong, Korea). Dimethyl sulfoxide (DMSO; ⩾99.9% purity) was purchased from BioShop Life Science (Burlington, ON, Canada). Tridemorph and desferriferrichrome were purchased from Cayman Chemical (Ann Arbor, MI, USA). Linoleyl alcohol and citicoline were purchased from Sigma-Aldrich (St. Louis, MO, USA). Nonadecanamide was purchased from Jiangsu Aikon (Jiangsu, China).

### 4.2. Collection and Culture of Nematode-Trapping Fungi and Nematodes

Nematode-trapping fungi (NTF) were isolated from 151 soil samples collected in Taiwan using the soil sprinkle method with some modification [37]. Briefly, soil samples were sprinkled on low nutrient medium (LNM,) and after 3–7 days, the plates were examined under microscopy and single conidia of the NTF were isolated and transferred to Potato Dextrose Agar (PDA) to establish pure cultures. The ITS regions were PCR amplified directly using fungal hyphae with the universal primers ITS1 (5’-TCCGTAGGTGAACCTGCGG-3’) and ITS4 (5’-TCCTCCGCTTATTGATATGC-3’). Species identity was assigned if the ITS sequence identity was 97% or higher. In total, 14 different species of NTF, including *Arthrobotrys oligospora,* were isolated from the 151 soil samples. For this study, 100 NTF strains of the genus *Arthrobotrys* were chosen, including 64 *Arthrobotrys oligospora* strains, 18 *Arthrobotrys thaumasia* strains, and 18 *Arthrobotrys musiformis* strains. These fungal strains were cultured on 100-mm LNM plates for 6 days at 25 °C as the control group. To induce the lifestyle switch to the predatory stage of the fungi, nematodes (*Caenorhabditis elegans*; details in preparation are described in the next section) were added uniformly on the 6-day old fungal cultures and co-cultivated for an additional 24 hours, which triggered the formation of the trapping devices.

Worms for control samples were prepared by placing 800~1000 synchronized nematodes embryos per NGM plate with bacteria (*Escherichia coli*) as the food source and collected at the adult stage for the extraction of metabolites. In total, roughly 10,000 nematodes (10 plates of worms) were harvested for metabolomic analyses as control samples. These control nematodes were never exposed to fungi. The worms used for the control and the fungi + worm plates were prepared in the same way as described. Well-fed nematodes were placed onto the fungal culture with no bacteria. These nematodes were captured by the fungi after 5–7 hours on the fungal culture and consumed by fungi by 24 hours when we collected the metabolites.

### 4.3. Extraction of Fungal Metabolites

For each strain, three agar-based cultures were prepared and combined for metabolite extraction. Fungi were scraped and collected with Falcon cell scrapers (Corning Inc., Corning, NY, USA), placed in a 2.0-mL microcentrifuge tube with 1 mL methanol, and then 500 μL ethyl acestate added. Extraction was facilitated with ultrasonication (Delta DC400; Heathrow Scientific, Vernon Hills, IL, USA) for 20 min. The extract was centrifuged at 12,000 rpm for 5 min by micro-centrifuge (Z216M; Hermle, Germany). Then the supernatant was transferred to another 2.0-mL microcentrifuge tube, and the pellet (mycelium) was kept for subsequent extraction with aqueous solvent. The supernatant was dried under vacuum (Vacufuge plus, Eppendorf; Germany), reconstituted with methanol in a ratio of 10.0 mg/mL. Second, the pellet was extracted by 1500 µl deionized water via the same process and finally reconstituted with deionized water in a ratio of 10.0 mg/mL. To sum up, the fungal cultures were sequentially extracted with organic solvent (methanol:ethyl acetate = 2:1, *v/v*) and aqueous solvent (water) to yield organic extracts and aqueous extracts, respectively. The control nematode samples were extracted by the same procedure. The extracts were stored at −80 °C before analysis.

### 4.4. Untargeted LC–MS Metabolomics

Untargeted metabolomics analysis was performed with a Q Exactive Plus mass spectrometer (Thermo Scientific, German) coupled with an ultra-high-performance liquid chromatography system (UltiMate 3000; Thermo, German). Each fungal extract was spiked with glycocholic acid (final conc. 100 nM) as the internal standard and subjected to analysis. The injection volume for each sample was 10 μL. A blank sample (methanol for organic extract; water for aqueous extract) was injected between every two samples to minimize carryover. LC configuration: The column oven temperature was set at 40 °C. Flow rate of mobile phase was set at 200 μL/min. A binary LC mobile phase system was applied: deionized water as solvent A and acetonitrile as solvent B, each containing 0.1% formic acid. The organic and aqueous extracts were separately analyzed with specified LC conditions. Organic extract was analyzed with a Syncronis C18 column (1.7 µm, 100 × 2.1 mm; Thermo Scientific, Waltham, MA, USA) under a 14-min gradient: 0–2.0 min 10% B; 2.0–8.0 min 10%–99% B; 8.0–10.0 min 99% B; 10.0–10.5 min 99%–10% B; 10.5–14.0 min 10% B. Aqueous extract was analyzed with a Syncronis HILIC column (1.7 µm, 100 × 2.1 mm; Thermo Scientific, U.S.A) under a 14-min gradient: 0–1.0 min 95% B; 1.0–8.0 min 10% B; 8.0–10.0 min 10% B; 10.0–10.5 min 95% B; 10.5–14.0 min 95% B. MS acquisition: A heated electrospray ionization (HESI) source was utilized for ionization, and the parameters of HESI were automatically optimized under a flow rate of 200 μL/min, giving capillary spray voltage at 3.5 kV, S-lens RF level at 50 (A.U.), capillary temperature at 275 °C, probe temperature at 400 °C, sheath gas at 30 (A.U.) and auxiliary gas at 10 (A.U.). Untargeted LC–MS metabolomics analysis was applied with a top-10 data-dependent acquisition (DDA) method. In each scanning segment, a full-MS1 spectrum was first acquired, followed by ten events of MS2 acquisition to the top-10 most intense ions in the MS1 spectrum. This segment was cycled throughout the analysis. All the data were acquired in positive ionization at *m*/*z* 100–1500. Spectral resolution was set at 70,000 for both MS1 and MS2 acquisition. Ion activation was facilitated by higher-energy collision-induced dissociation (HCD) with stepped normalized collision energy (NCE) of 20, 30, and 40 (A.U.).

### 4.5. Metabolomics Analysis With GNPS Molecular Networking

To create molecular networks, LC–MS .raw data files were converted into .mzXML format with MSConvert (ProteoWizard, http://proteowizard.sourceforge.net) and processed on GNPS (http://gnps.ucsd.edu). The molecular networks were created using the GNPS online workflow (METABOLOMICS-SNETS-V2, version release_14). The data were filtered by removing all MS/MS fragment ions within +/− 17 Da of the precursor *m*/*z*. The precursor ion mass tolerance was set to 0.02 Da and an MS/MS fragment ion tolerance of 0.5 Da. A network was then created where edges were filtered to have a cosine score above 0.65 and more than 4 matched peaks. Further, edges between two nodes were kept in the network if and only if each of the nodes appeared in each other’s respective top 10 most similar nodes. Finally, the maximum size of a molecular family was set to 500, and the lowest-scoring edges were removed from molecular families until the molecular family size was below this threshold. The spectra in the network were then searched against GNPS spectral libraries. The library spectra were filtered in the same manner as the input data. All matches kept between network spectra and library spectra were required to have a score above 0.65 and at least 4 matched peaks. Cytoscape 3.4.0 was used for visualization of molecular networks.

The two datasets acquired from the organic extract and the aqueous extract were separately processed with the above parameters to create two molecular networks (Figure 2 and Figure S1 for organic extract and aqueous extract, respectively). As a result, the molecular network of the organic extract (or the aqueous extract) yielded 4570 (5553) nodes detected in the fungal extracts (nodes detected in blank controls were excluded), among which 682 (858) nodes were detected in the saprophytic stage, 706 (2032) nodes were detected in the predatory stage, and 3182 (2663) nodes were detected in both stages; together, the two molecular networks gave 10,123 nodes detected in the fungal extracts. The molecular networks of the organic extract and aqueous extract were publically available at https://gnps.ucsd.edu/ProteoSAFe/status.jsp?task=06353598018844edbdc8e411496c57f6 and https://gnps.ucsd.edu/ProteoSAFe/status.jsp?task=15c3501c84904de285cd2fed571972aa, respectively.

### 4.6. Quantitative Analysis and Identification of NTF Metabolites

The metabolomics data was processed by Compound Discoverer (CD) (version 2.1; Thermo, Waltham, MA, USA) for quantitative analysis in order to sort out fungal metabolites significantly enriched in the predatory stage. CD enabled picking chemical features with the following parameters: mass tolerance of 5 ppm, minimal peak intensity of 10,000, and alignment minute tolerance of 0.2 min. Chemical features that had significant fold-changes particularly in the predatory stage were sequentially sorted out by the following procedures: (i) chemical features with averaged peak area less than 100,000 in the blank samples were selected, (ii) chemical features with averaged peak area 10-fold higher in the samples extracted from the predatory stage of the NTF than in the blank sample were selected, (iii) chemical features with averaged peak area 10-fold higher in the samples extracted from the predatory stage of the NTF than from the saprophytic stage of the NTF were selected, (iv) chemical features with averaged peak area significantly enriched (adjusted *p*-value <0.05) in the samples extracted from the predatory stage of the NTF than from the saprophytic stage of the NTF were selected. For structural identification, the selected features having a higher frequency of detection in the strains in the predatory stage than in the saprophytic stage (i.e., the blue nodes being detected more frequently in the samples in the predatory stage than in the saprophytic stage) were initially sorted out. Among those sorted compounds, the top-100 intense compounds in the organic and aqueous datasets were selected for structural identification via searching against GNPS spectral libraries, respectively. The given annotations were validated by LC–MS/MS examination of their chemical standards. Eventually, three metabolites (linoleyl alcohol, nonadecanamide, and desferriferrichrome) in the organic extract and one metabolite (citicoline) in the aqueous extract were identified. The metabolite identification reported in this study sticks to the reporting standards for metabolomics experiments announced by Chemical Analysis Working Group (CAWG) Metabolomics Standards Initiative (MSI) [38]. The lists of the identified metabolites in this study are summarized in the supplementary material.

### 4.7. Bioactivity Test

To test the activities of the standard target compounds, 30 μL of the compounds of desired concentration was added onto the nematode growth media (NGM) medium in 3.5-cm Petri dishes. Subsequently, the compounds were spread uniformly until dried. Next, OP50 (*E. coli*) was added onto the plate as the food source for *C. elegans*. Then, 30~35 synchronized nematodes embryos were grown on these plates containing the compounds and their growth was monitored closely monitored for the next ~3 days with a stereoscopic microscope (Stemi 305; ZEISS, Oberkochen, Germany) and stand-alone camera (Axiocam ERc 5s; ZEISS, Oberkochen, Germany). The effects of the compounds were mainly assessed by the growth and the development of the embryos to reach adulthood compared to the solvent control.

### 4.8. Data Availability

The LC–MS metabolomics dataset in this study has been deposited to the EMBL-EBI MetaboLights database [39] with the identifier MTBLS1515 and can be accessed at https://www.ebi.ac.uk/metabolights/MTBLS1515. The other data that support the findings of this study are available from the corresponding author upon reasonable request.

The tables of MS/MS spectral lists of the uncharacterized metabolites, particularly detected and enriched in the predatory stage, are supplemented as appendixes. The metabolites detected in the organic or aqueous extract are supplemented as Appendix A and Appendix B, respectively.

## Supplementary Materials

The following are available online at https://www.mdpi.com/2218-1989/10/3/117/s1. Figure S1: Molecular networking of the aqueous metabolic extracts of the 100 *Arthrobotrys* strains during the fungi-nematode interaction. Figure S2: Tandem mass spectrometric interrogation of the singly-charged *Arthrobotrys musiformis* trap-associated peptides (AmTPs). Figure S3: Tandem mass spectral comparison between desferriferrichrome and its deoxygenated analogue. Figure S4: Validation of the identified fungal metabolite—linoleyl alcohol. Figure S5: Validation of the identified fungal metabolite—nonadecanamide. Figure S6: Validation of the identified fungal metabolite—citicoline. Figure S7: The abundance of the citicoline in each *Arthrobotrys* strain. Figure S8: Comparison of the MS/MS spectra of the uncharacterized fungal metabolites in the organic extracts with those in the public metabolomics datasets extracted by MASST. Figure S9: Comparison of the MS/MS spectra of the uncharacterized fungal metabolites in the aqueous extracts with those in the public metabolomics datasets extracted by MASST. Table S1: List of the 100 *Arthrobotrys* strains isolated in Taiwan. Table S2: List of the putatively characterized *Arthrobotrys musiformis* trap-associated peptides (AmTPs). Table S3: List of the characterized desferriferrichrome analogues produced by *Arthrobotrys* fungi. Table S4: List of the identified metabolites produced by *Arthrobotrys* fungi in the predatory stage. Table S5: The predicted molecular formula of the uncharacterized metabolites enriched in the organic extracts in the predatory stage of NTF using SIRIUS 4.0. The list of the MS/MS spectral peaks is provided in Appendix A. Table S6: Linking the uncharacterized (organic) metabolites with the sample information in public metabolomics datasets by MASST. Table S7: The predicted molecular formula of the uncharacterized metabolites enriched in the aqueous extracts in the predatory stage of NTF using SIRIUS 4.0. The list of the MS/MS spectral peaks is provided in Appendix B. Table S8: Linking the uncharacterized (aqueous) metabolites with the sample information in public metabolomics datasets by MASST.

## Appendix A

The MS2 peak lists of the uncharacterized metabolites particularly detected and enriched in the predatory stage in the organic extract.

**Table A1:** The MS2 peak lists of the uncharacterized metabolites detected commonly in *A. oligospora*, *A. thaumasia*, and *A. musiformis*.

**[M+H]** ***m*/*z* 454.229**		**[M+H]** ***m*/*z* 376.144**		**[M+H]** ***m*/*z* 519.193**		**[M+H]** ***m*/*z* 462.208**		**[M+H]** ***m*/*z* 590.23**		**[M+H]** ***m*/*z* 726.281**		**[M+H]** ***m*/*z* 293.276**	
***m*/*z***	**Relative Intensity**	***m*/*z***	**Relative Intensity**	***m*/*z***	**Relative Intensity**	***m*/*z***	**Relative Intensity**	***m*/*z***	**Relative Intensity**	***m*/*z***	**Relative Intensity**	***m*/*z***	**Relative Intensity**
110.07	11.61	57.07	1.62	72.08	15.06	60.05	24.86	72.08	44.2	65.14	0.97	56.05	4.93
156.08	9.8	69.07	6.42	84.04	24.18	70.07	12.95	84.04	21.28	65.14	3.3	57.07	3.33
166.06	10.09	72.08	3.18	88.04	100	84.04	15.17	88.04	83.5	70.07	0.58	58.07	4.44
196.13	6.19	81.07	2.41	91.06	9.05	86.1	7.72	102.06	86.46	86.1	0.29	71.09	1.91
212.16	5.64	83.09	2.05	102.06	73.8	96.04	10.21	130.05	19.95	97.56	0.28	74.06	6.37
231.15	4.45	86.1	8.99	118.09	12.04	97.03	6.18	158.04	53.11	565.21	0.31	75.06	2.32
249.16	100	95.09	1.89	130.05	29.73	98.06	9.01	185.06	17.05	579.23	0.23	88.08	5.04
250.15	4.38	120	2.56	158.04	45.8	116.07	94.24	188.07	3.73	606.24	0.74	89.08	2.6
250.16	10.61	120.1	7.36	167.06	11.96	119.09	20.38	189.12	11.48	608.25	0.3	118.09	1.74
255.17	20.37	144	5.73	181.06	7.06	120.09	12.2	217.08	14.18	610.21	0.26	122.08	3.34
276.17	33.82	157.1	2.12	183.11	13.45	126.05	65.61	227.07	6.37	623.22	1.07	123.08	2.47
305.15	12.93	163.1	2.47	184.07	13.44	127.04	6.27	245.08	100	624.22	0.38	124.09	3.11
321.23	11.79	166.1	1.59	185.06	20.54	135.08	5.59	246.08	3.92	666.26	4.72	242.25	6.44
322.18	93.05	177.1	4.68	217.08	21.39	138.05	51.83	255.06	4.11	667.27	1.69	243.25	5.65
323.18	10.95	207.1	4.85	229.12	22.28	144.07	69.8	273.07	53.79	712.28	0.41	244.25	23.1
393.21	9	259.1	1.81	245.08	68.71	168.07	17.13	374.12	6.93	725.27	0.93	245.26	2.09
410.24	89.14	375.4	3.72	273.07	36.49	186.08	72.9	384.1	5.27	726.28	100	292.27	100
411.24	11.98	376.1	100	299.06	21.93	204.09	100	402.11	62.56	727.28	22.3	293.28	20.39
454.23	49.59	377.2	18.14	344.14	6.37	305.03	5.16	403.12	5.49	735.83	0.24	293.29	2.07
455.23	7.81	377.3	5.29	402.11	8.41	347.14	5.82	501.18	5.61	738.61	0.2	294.28	5.76
**[M+H]** ***m*/*z* 317.191**		**[M+H]** ***m*/*z* 372.311**		**[M+H]** ***m*/*z* 688.326**		**[M+H]** ***m*/*z* 768.539**		**[M+2H]** ***m*/*z* 376.145**		**[M+H]** ***m*/*z* 529.409**		**[M+H]** ***m*/*z* 184.606**	
***m*/*z***	**Relative Intensity**	***m*/*z***	**Relative Intensity**	***m*/*z***	**Relative Intensity**	***m*/*z***	**Relative Intensity**	***m*/*z***	**Relative Intensity**	***m*/*z***	**Relative Intensity**	***m*/*z***	**Relative Intensity**
69.07	6.11	67.05	23.85	65.22	13.43	69.07	13.05	57.07	2.51	57.07	10.97	60.08	5.03
70.07	9.3	69.07	31.65	70.07	11.57	71.09	6.61	69.07	9.72	67.05	8.06	86.1	20.62
72.08	100	71.09	52.72	86.06	85.99	81.07	12.78	72.08	5.09	69.07	7.42	95.06	5.27
74.06	6.13	72.08	23.47	113.07	20.09	83.09	16.59	81.07	3.39	70.07	10.95	108.08	13.07
86.06	30.19	81.07	50.84	114.06	11.62	86.1	11.62	83.09	2.97	71.05	16.6	110.07	75.47
86.1	27.21	83.09	50.58	127.09	22.25	95.09	14.21	86.1	9.58	71.09	8.26	112.04	6.53
87.04	10.43	86.1	91.67	128.07	54.43	97.1	18.65	95.09	2.55	72.08	38.37	117.98	4.69
104.07	39.82	95.09	48.82	131.08	68.37	107.09	8.48	120.08	6.65	81.07	22.06	125	10.85
136.08	10	97.1	67.3	145.1	26.85	109.1	9.18	157.1	3.1	84.08	6.72	131.59	10.63
173.13	6.72	98.06	30.11	173.09	31.49	111.12	9.99	163.07	7.07	86.1	80.38	132.08	9.37
186.08	28.5	99.08	48.21	188.1	27.67	119.09	100	177.09	13.41	95.09	100	136.08	13.1
200.09	9.53	107.09	22.87	214.08	14.98	120.09	7.67	207.1	13.88	120.08	18.97	152.6	19.85
201.12	9.93	109.1	25.99	230.11	16.93	135.08	25.38	269.14	2.37	123.08	38.29	153.1	100
219.13	10.51	121.1	24.21	287.13	18.35	184.07	86.22	283.15	2.41	141.09	26.44	154.11	8.56
232.14	9.22	125.1	55.11	302.15	11.01	185.08	9.87	375.3	4.42	159.1	55.03	156.08	57.32
260.14	7.84	189.11	67.43	344.16	29.83	212.2	6.58	375.33	2.35	173.12	23.28	161.6	12.15
316.19	6.39	238.25	21.94	345.18	21.88	319.3	12.97	376.14	100	184.07	7.27	166.1	11.22
316.2	60.68	277.22	24.3	516.24	27.3	336.33	12.52	376.25	3.34	283.26	13.97	184.07	64.13
317.13	9.87	280.26	66.94	688.33	100	354.34	15.36	377.15	17.36	313.27	65.21	184.11	4.52
317.2	20.15	294.24	100	689.33	21.33	355.32	7.48	377.32	6.1	341.27	8.24	308.17	8.36

**Table A2:** The MS2 peak lists of the uncharacterized metabolites detected specifically in *A. oligospora*.

[M+H] *m*/*z* 209.128	
*m*/*z*	Relative Intensity
56.05	8.09
70.07	6.79
80.05	23.65
84.08	100
107.09	3.52
120.08	3.59
121.62	4.15
130.09	55.04
135.12	3.37
147.06	6.36
153.09	4.2
175.05	3.85
192.08	9.72
203.94	4.47
204.97	4.13
209.06	3.43
209.1	3.25
209.13	26.17
209.15	10.25
226.95	3.5

**Table A3:** The MS2 peak lists of the uncharacterized metabolites detected specifically in *A. thaumasia*.

[M+H] *m*/*z* 216.042		[M+H] *m*/*z* 259.992		[M+H] *m*/*z* 338.171		[M+H] *m*/*z* 339.249		[M+H] *m*/*z* 368.182		[M+H] *m*/*z* 423.005		[M+H] *m*/*z* 443.287		[M+2H] *m*/*z* 344.662	
*m*/*z*	Relative Intensity	*m*/*z*	Relative Intensity	*m*/*z*	Relative Intensity	*m*/*z*	Relative Intensity	*m*/*z*	Relative Intensity	*m*/*z*	Relative Intensity	*m*/*z*	Relative Intensity	*m*/*z*	Relative Intensity
70.07	7.5	70.07	16.21	69.07	2.71	58.07	0.47	57.07	25.69	70.03	100	70.07	50.76	57.07	11.17
72.08	16.1	72.08	31.36	70.07	9.57	59.07	0.1	70.07	81.62	70.07	29.12	72.08	100	70.07	38.89
84.04	5	84.04	21.93	72.08	8.7	60.08	1.15	72.08	64.89	72.08	21.37	74.06	5.76	71.09	11.23
86.1	2.74	84.08	33.27	81.07	2.78	70.07	0.12	74.06	57.6	74.02	17.66	84.04	8.47	72.08	33.61
116.07	20.21	86.1	71	84.04	3.6	84.08	0.07	84.04	27.38	74.06	5.61	84.08	11.12	84.04	11.02
129.07	4.51	115.09	9.04	84.08	4.54	86.1	0.08	84.08	29.26	84.04	8.23	86.1	76.85	86.06	100
130.05	20.18	116.07	20.55	86.1	21.37	95.06	0.08	86.1	100	84.08	13.13	98.98	6.12	86.1	43.92
134.06	3.41	126.05	9.66	110.07	2.4	104.07	0.14	110.07	26.36	86.1	36.16	100.08	12.28	87.06	17.66
135.07	3.26	126.09	7.41	113.07	6.87	104.11	100	112.08	22.14	88.04	7.25	110.07	9.15	100.04	13.69
147.08	5.15	129.1	10.25	115.09	6.99	104.14	0.09	113.07	27.09	102.06	4.38	120.08	10.91	113.07	21.62
153.01	5.74	132.1	14.99	120.04	9.3	105.1	0.17	120.08	86	104.11	4.9	128.11	81.72	114.06	30.06
155.07	2.54	135.07	18.18	120.08	100	105.11	3.08	121.03	38.73	110.07	7.63	129.1	9.46	115.05	19.51
157	17.74	143.12	8.76	121.08	5.53	110.07	0.16	130.09	78.38	120.08	14.81	136.08	8.86	116.07	16.69
170.04	100	185.13	7.07	129.1	2.98	112.9	0.27	136.08	57.1	126.09	5.53	145.13	9.1	126.09	11.16
171.04	5.8	186.11	8.94	143.08	4	114.89	0.07	157.1	22.37	129.1	8.25	199.18	20.37	127.09	13.31
181	9.86	189.13	7.45	166.09	7.52	156.04	0.2	166.09	45.91	130.05	5.16	200.09	9.25	128.07	21.61
199.02	25.56	197.13	12.81	191.12	47.59	156.08	0.13	175.11	81.37	135.07	17.89	216.17	23.44	131.08	91.89
215.14	2.63	200.95	22.27	192.12	3.9	192.02	0.34	203.1	60.27	136.08	8.14	216.21	32.01	173.09	16.18
216.04	8.76	213.99	100	219.11	15.14	193	0.18	221.13	28.81	156.08	4.42	217.12	5.35	188.1	18.46
217.1	5.68	224.95	12.12	237.12	11.49	207.01	0.15	223.11	79.2	164.05	16.17	229.15	11.2	228.13	15.99

**Table A4:** The MS2 peak lists of the uncharacterized metabolites detected specifically in *A. musiformis*.

**[M+H]** ***m*/*z* 250**		**[M+H]** ***m*/*z* 262**		**[M+H]** ***m*/*z* 271.2**		**[M+H]** ***m*/*z* 323.2**		**[M+H]** ***m*/*z* 354.24**		**[M+H]** ***m*/*z* 361.217**	
***m*/*z***	**Relative Intensity**	***m*/*z***	**Relative Intensity**	***m*/*z***	**Relative Intensity**	***m*/*z***	**Relative Intensity**	***m*/*z***	**Relative Intensity**	***m*/*z***	**Relative Intensity**
67.05	5.04	60.05	7.12	57.07	14.55	69.07	12.39	58.07	9.21	57.07	6.46
70.03	10.16	70.07	7.91	69.07	16.54	81.07	7.86	60.05	4.03	69.07	3.67
71.09	4.95	72.08	10.21	70.07	81.26	93.07	5.07	70.07	9.36	71.09	5.71
74.02	4.82	84.04	7.05	71.09	16.45	95.09	7.17	72.08	10.83	72.08	32.84
81.07	7.33	86.1	50.12	72.08	100	105.07	9.21	74.06	5.25	73.08	9.9
86.1	32.91	87.1	5.7	84.08	17.44	107.09	4.84	84.08	4.72	85.1	3.7
87.1	8.57	102.1	7.17	86.1	54.97	109.1	8.16	86.1	9.09	86.1	11.01
88.04	5.13	130.1	8.47	112.08	33.05	121.1	4.8	91.06	5.39	95.09	4.08
95.09	8.69	132.1	12.91	112.09	14.26	123.12	5.28	98.06	4.4	97.08	4.9
109.1	5.21	135.1	23.16	113.07	13.47	145.1	4.68	116.07	20.31	114.1	49.09
132.1	8.07	148.1	5.42	115.09	45.92	157.1	5.82	120.08	17.95	115.1	3.44
146.06	8.44	156.1	8.46	140.07	32.89	197.1	4.91	143.08	10.34	115.11	20.67
190.97	8.99	199	7.13	140.08	19.24	211.11	5.61	152.06	7	116.11	3.6
201.12	7.32	203	23.05	141.07	15.82	239.11	4.98	161.09	16.68	200.09	7.72
203.13	7.06	216	100	225.16	18.69	277.19	5.19	166.09	14.44	201.09	12.74
204	100	217	6.93	254.14	22.68	295.21	30.34	189.09	13.91	202.1	10.02
205	5.87	227	11.29	254.17	30.03	296.21	4.59	241.15	14.31	301.17	3.79
232.98	6.91	245	27.71	271.18	23.19	305.19	10.95	354.16	4.92	342.31	4.37
249.13	7.59	262	14.2	271.2	17.09	323.2	100	354.24	100	360.21	100
250	10.76	262.1	10.83	272.15	26.07	324.2	14.23	355.24	12.38	361.22	21.86
**[M+H]** ***m*/*z* 373.244**		**[M+2H]** ***m*/*z* 390.099**		**[M+H]** ***m*/*z* 443.287**		**[M+H]** ***m*/*z* 519.265**		**[M+H]** ***m*/*z* 550.446**		**[M+H]** ***m*/*z* 945.327**	
***m*/*z***	**Relative Intensity**	***m*/*z***	**Relative Intensity**	***m*/*z***	**Relative Intensity**	***m*/*z***	**Relative Intensity**	***m*/*z***	**Relative Intensity**	***m*/*z***	**Relative Intensity**
69.07	4.02	57.07	11.27	69.07	2.18	60.05	7.51	57.07	3.63	70.07	2.87
70.07	15.54	65.16	8.11	70.07	15.57	70.07	9.88	67.05	1.73	112.08	2.36
72.08	100	70.07	16.05	72.08	12.8	72.08	12.22	69.07	2.98	199.02	1.36
84.08	4.86	71.09	18.33	84.08	3.61	84.04	6.04	71.09	2.83	201.03	2.51
86.1	15.77	72.08	6.77	86.1	16.19	104.11	7.01	81.07	2.96	370.09	5.8
91.06	14.44	74.06	2.1	100.08	4.07	116.07	100	83.09	2.15	397.07	3.81
112.08	4.06	84.04	2.21	120.08	2.19	126.06	20.27	85.1	1.61	398.07	2.07
112.11	3.93	84.08	3.31	128.11	100	128.11	5.53	86.06	2.1	398.09	9.92
116.07	21.32	86.1	18.32	129.11	5.29	138.05	17.41	87.04	12.16	427.11	2.08
173.09	21.63	105.07	4.77	132.03	3.64	144.07	21.64	95.09	3.24	455.11	4.93
187.11	4.79	112.08	3.81	142.12	8	167.06	4.99	104.11	2	484.14	1.37
200.09	39.96	128.12	5.15	199.18	2.83	168.07	5.21	109.1	1.71	512.13	3.94
285.14	56.56	129.1	2.84	200.09	17.91	173.13	16.19	114.09	2.6	541.16	6.02
286.15	5.01	149.02	100	211.14	2.22	184.07	15.16	184.07	5.94	569.15	19.36
302.17	25.25	167.03	14.47	216.21	3.3	186.08	20.9	219.17	100	570.16	2.45
356.22	63.4	201.03	3.3	226.19	2.31	201.12	27.99	220.18	8.14	586.18	2.26
357.22	6.39	218.1	3.63	229.15	3.74	204.09	24.9	236.15	2.39	713.24	5.58
373.22	5.6	247.04	3.08	285.14	20.71	217.12	6.46	256.26	6.9	723.23	2.15
373.24	78.97	258.23	8.17	286.15	2.25	299.06	9.06	277.27	1.71	741.24	100
374.25	8.79	370.09	2.5	356.22	2.03	316.19	15.78	305.21	10.37	742.24	19.74

## Appendix B

The MS2 peak lists of the uncharacterized metabolites particularly detected and enriched in the predatory stage in the aqueous extract.

**Table A5:** The MS2 peak lists of the uncharacterized metabolites detected commonly in *A. oligospora, A. thaumasia,* and *A. musiformis*.

***m*/*z* 413.276** **[M+H]**		***m*/*z* 750.558** **[M+H]**		***m*/*z* 314.207** **[M+H]**		***m*/*z* 455.189** **[M+H]**		***m*/*z* 484.318** **[M+H]**		***m*/*z* 498.328** **[M+H]**		***m*/*z* 528.302** **[M+H]**	
***m*/*z***	**Relative Intensity**	***m*/*z***	**Relative Intensity**	***m*/*z***	**Relative Intensity**	***m*/*z***	**Relative Intensity**	***m*/*z***	**Relative Intensity**	***m*/*z***	**Relative Intensity**	***m*/*z***	**Relative Intensity**
70.07	100.00	53.00	0.37	70.07	66.12	70.07	80.81	70.07	100.00	70.07	100.00	70.07	100.00
72.08	5.78	57.07	0.91	71.07	2.18	72.08	6.66	72.08	33.04	72.08	47.48	72.08	17.11
86.10	34.36	71.09	12.07	72.08	25.07	86.10	23.27	86.10	75.91	86.10	30.67	86.10	36.53
141.10	19.95	72.09	0.31	86.10	25.57	112.05	74.89	120.08	16.10	116.07	11.04	132.10	5.93
169.10	25.46	86.10	0.43	97.03	7.25	120.08	7.94	136.06	18.74	120.08	7.16	137.05	43.24
169.13	6.44	88.11	100.00	98.06	1.38	136.08	8.58	137.05	81.88	137.05	36.15	155.08	22.53
183.15	20.40	89.12	2.73	136.06	15.04	141.10	17.40	141.10	34.31	143.12	13.32	169.13	10.14
197.13	6.89	327.08	0.54	137.05	2.82	169.10	23.64	155.08	10.22	155.08	18.15	183.15	20.99
203.14	5.66	383.14	2.37	169.13	12.06	183.15	7.95	169.10	60.80	169.13	12.85	187.07	7.86
211.14	19.26	439.20	6.07	173.13	7.58	211.14	7.55	169.13	14.20	171.11	12.32	197.13	17.04
212.03	7.89	440.21	0.91	197.13	14.13	215.14	55.14	183.15	30.28	183.15	13.32	211.14	25.60
212.96	6.53	495.27	11.15	201.12	5.67	216.14	8.35	197.13	18.12	197.13	14.23	243.13	13.07
228.01	17.04	496.27	2.14	215.14	100.00	229.15	14.15	203.14	10.32	211.14	15.63	284.12	20.52
229.15	33.77	551.33	5.12	216.14	7.28	454.21	43.52	211.14	22.77	229.15	92.38	286.11	7.27
282.18	5.73	552.33	1.17	223.02	2.35	454.27	8.37	215.14	18.01	230.16	8.54	310.21	13.22
300.19	20.49	607.39	4.46	226.99	5.35	455.19	100.00	229.15	11.73	236.15	6.99	397.21	9.25
329.03	7.05	608.39	1.17	314.06	1.52	455.21	22.15	282.18	28.30	268.17	10.35	415.22	23.04
345.00	26.35	663.45	4.69	314.09	2.29	455.25	7.12	300.19	17.27	328.22	5.82	528.22	5.95
413.14	7.87	664.46	1.31	314.21	20.46	456.19	12.23	395.26	15.04	498.33	14.44	528.30	30.11
413.28	17.60	750.56	0.34	315.21	1.96	456.28	6.95	484.31	25.52	498.38	6.56	528.34	8.45
***m*/*z* 565.167** **[M+H]**		***m*/*z* 145.170** **[M+H]**		***m*/*z* 422.065** **[M+H]**		***m*/*z* 356.213** **[M+H]**		***m*/*z* 418.167** **[M+H]**		***m*/*z* 647.193** **[M+H]**	
***m*/*z***	**Relative Intensity**	***m*/*z***	**Relative Intensity**	***m*/*z***	**Relative Intensity**	***m*/*z***	**Relative Intensity**	***m*/*z***	**Relative Intensity**	***m*/*z***	**Relative Intensity**
70.07	2.05	58.07	0.30	58.07	24.79	58.07	3.18	60.06	5.77	58.07	1.45
72.08	0.99	60.06	2.36	70.07	15.51	69.07	5.12	70.07	24.03	70.07	2.65
81.03	17.41	60.08	11.09	72.08	6.34	70.07	10.99	72.08	4.31	72.08	1.08
82.04	0.50	69.07	3.49	84.08	19.46	72.08	100.00	84.08	8.70	77.01	1.67
84.04	0.73	70.07	3.43	86.10	100.00	73.08	2.72	86.10	8.23	84.08	3.75
84.08	1.20	72.08	0.51	218.98	6.38	84.08	9.70	95.02	9.48	86.10	3.24
86.10	1.62	84.08	1.17	284.05	4.88	86.10	11.31	110.07	25.38	95.02	5.00
110.07	0.72	86.10	100.00	318.07	16.13	110.07	4.47	112.09	7.50	117.04	1.06
129.10	0.94	87.09	0.23	322.03	18.65	112.11	2.63	129.10	16.63	129.10	1.85
136.06	100.00	87.10	3.84	326.09	8.05	129.10	2.40	168.07	4.11	160.97	1.11
137.06	1.01	112.09	0.29	370.03	42.32	132.03	9.35	175.12	4.10	168.07	2.30
137.07	2.86	116.07	1.71	371.03	10.69	144.03	3.91	199.01	5.99	199.01	3.82
161.06	0.94	121.62	0.44	376.06	5.80	156.08	2.99	253.09	4.93	244.07	2.94
216.09	7.03	121.97	0.27	393.05	5.34	200.09	39.67	268.10	18.53	268.10	17.27
234.10	0.96	130.09	0.26	394.07	32.52	211.14	4.11	286.11	100.00	286.11	100.00
286.11	2.66	130.10	0.62	395.07	8.85	285.14	66.27	287.12	6.04	287.12	4.33
332.07	23.31	144.62	0.69	412.08	7.77	286.15	5.89	401.18	4.03	288.11	1.10
333.08	1.16	145.17	8.02	422.06	34.08	328.09	3.14	418.20	23.30	324.07	6.70
412.10	0.97	146.17	0.46	423.06	10.78	356.22	33.52	419.19	5.39	424.10	1.04
430.11	2.99	158.09	0.53	440.07	13.11	357.22	3.70	419.21	4.38	624.13	2.78

**Table A6:** The MS2 peak lists of the uncharacterized metabolites detected specifically in *A. oligospora*.

*m*/*z* 485.61 [M+2H]		*m*/*z* 461.605 [M+2H]		*m*/*z* 423.172 [M+H]		*m*/*z* 227.033 [M+H]		*m*/*z* 299.543 [M+2H]		*m*/*z* 557.15 [M+H]	
*m*/*z*	Relative Intensity	*m*/*z*	Relative Intensity	*m*/*z*	Relative Intensity	*m*/*z*	Relative Intensity	*m*/*z*	Relative Intensity	*m*/*z*	Relative Intensity
70.07	2.55	70.07	1.96	60.05	39.12	56.05	6.60	58.07	10.05	70.07	18.93
84.08	4.14	84.08	2.12	74.06	15.31	70.07	2.38	59.07	3.47	72.08	4.35
86.10	2.45	86.10	1.93	84.04	6.56	76.04	100.00	64.81	5.41	81.03	35.64
95.02	1.37	97.03	24.82	84.08	9.19	77.04	1.44	84.08	4.97	84.04	3.49
97.03	2.54	111.04	3.90	88.04	18.53	84.04	0.85	86.10	4.23	84.08	10.79
112.05	100.00	112.05	100.00	102.06	6.26	84.08	1.10	117.04	2.87	86.10	8.83
113.05	3.41	113.05	3.52	120.07	38.68	106.00	6.99	136.06	3.16	88.04	2.93
129.10	2.27	175.12	8.76	126.06	7.30	110.07	0.66	197.00	12.14	110.07	3.91
136.08	3.44	176.12	2.38	130.05	10.30	116.07	1.14	256.07	9.91	112.05	34.75
152.07	3.76	180.09	40.43	144.07	44.50	124.01	76.85	268.10	29.65	120.08	2.57
154.09	4.63	181.09	2.53	153.07	5.56	125.01	1.15	270.04	57.43	129.10	6.41
205.06	2.47	193.05	2.21	171.08	42.90	153.03	0.72	270.54	10.02	152.06	100.00
223.07	4.82	208.07	3.46	189.09	100.00	181.03	34.17	271.04	5.07	153.06	3.13
286.11	4.48	286.11	8.54	190.09	5.28	181.06	0.75	289.99	5.72	161.06	7.55
303.04	1.67	287.12	4.06	199.07	45.80	182.03	1.16	298.55	2.78	268.10	7.76
321.05	2.84	288.11	3.44	207.10	57.59	209.02	6.84	299.55	100.00	270.10	3.71
340.03	1.58	309.04	1.84	217.08	17.48	209.13	1.42	300.05	21.83	286.11	27.18
357.04	2.28	420.08	2.57	272.12	5.38	226.15	1.22	300.54	7.37	311.20	2.69
358.04	3.48	461.23	2.46	286.10	21.43	227.03	18.21	308.00	7.28	348.07	6.19
616.09	1.27	462.01	2.23	387.15	12.65	228.04	0.80	336.07	3.14	446.11	3.61

**Table A7:** The MS2 peak lists of the uncharacterized metabolites detected specifically in *A. thaumasia*.

***m*/*z* 228.05** **[M+H]**		***m*/*z* 364.186** **[M+H]**		***m*/*z* 425.214** **[M+H]**		***m*/*z* 439.291** **[M+H]**		***m*/*z* 470.297** **[M+H]**		***m*/*z* 512.344** **[M+H]**		***m*/*z* 524.198** **[M+H]**		***m*/*z* 528.339** **[M+H]**	
***m*/*z***	**Relative Intensity**	***m*/*z***	**Relative Intensity**	***m*/*z***	**Relative Intensity**	***m*/*z***	**Relative Intensity**	***m*/*z***	**Relative Intensity**	***m*/*z***	**Relative Intensity**	***m*/*z***	**Relative Intensity**	***m*/*z***	**Relative Intensity**
69.07	5.24	70.07	100.00	70.07	33.28	70.07	100.00	70.07	100.00	70.07	17.78	70.07	37.47	70.07	100.00
70.07	38.05	72.08	7.86	72.08	4.78	86.10	39.92	72.08	15.36	72.08	3.17	72.08	22.17	72.08	42.51
72.08	6.57	74.06	9.28	86.10	21.60	98.06	7.23	86.10	53.40	86.10	31.06	84.04	28.51	86.10	50.14
74.02	68.32	86.10	8.31	136.08	6.31	99.08	12.62	137.05	89.26	112.05	100.00	84.08	44.53	137.05	68.47
84.04	6.20	120.08	30.70	169.13	10.14	116.07	12.67	141.10	16.30	113.05	3.08	86.10	49.10	155.08	9.86
84.08	20.30	166.09	7.85	183.15	4.14	121.06	14.55	169.10	26.85	116.07	3.23	87.06	13.53	169.13	29.28
86.10	17.95	168.07	6.72	197.13	8.95	126.05	7.29	169.13	7.23	130.10	4.88	88.04	23.74	183.15	30.33
109.03	4.54	171.11	7.11	211.14	5.02	138.09	9.20	183.15	5.88	137.05	73.35	102.06	27.32	197.13	35.50
110.07	8.99	172.99	9.11	215.14	3.84	159.10	4.78	187.11	21.32	143.12	4.97	110.07	24.31	211.14	46.04
129.10	12.54	195.98	8.17	229.15	5.61	183.15	52.35	197.13	10.84	155.08	5.56	120.08	82.38	229.15	10.12
136.04	22.94	199.11	9.68	233.13	6.30	184.15	3.76	203.14	7.73	171.11	9.36	129.10	52.55	243.13	8.70
148.98	5.36	217.12	52.56	263.14	5.78	211.14	67.36	211.14	4.49	183.15	3.08	166.09	100.00	284.12	8.03
155.03	77.72	217.13	11.88	312.15	6.84	212.15	5.11	214.15	4.10	211.14	3.64	181.06	22.08	286.11	21.72
156.04	4.36	245.13	9.35	326.21	5.41	229.15	37.89	229.15	71.45	229.15	6.44	199.07	14.12	310.21	32.24
156.08	4.19	245.98	6.43	354.16	3.91	324.23	11.61	230.16	4.33	244.09	8.12	217.08	73.26	415.22	9.90
182.04	100.00	263.14	16.43	425.20	9.48	326.21	33.32	245.11	8.40	245.19	5.10	223.11	20.85	415.25	9.10
183.05	6.15	346.08	8.62	425.22	100.00	327.21	4.64	268.17	11.39	268.17	4.83	245.08	62.37	423.30	8.99
211.02	5.99	364.09	9.45	425.24	11.34	439.23	14.52	300.19	10.40	284.20	8.97	280.13	37.69	528.28	8.08
228.05	11.02	364.19	18.72	425.27	4.51	439.29	24.98	381.25	4.37	399.26	2.97	302.10	27.16	528.30	11.75
228.23	14.22	365.18	5.85	426.22	14.76	440.29	4.56	470.30	9.64	512.34	4.88	359.12	26.94	528.34	26.31
***m*/*z* 532.144** **[M+H]**		***m*/*z* 567.35** **[M+H]**		***m*/*z* 599.34** **[M+H]**		***m*/*z* 617.182** **[M+H]**		***m*/*z* 618.155** **[M+H]**		***m*/*z* 347.653** **[M+2H]**		***m*/*z* 529.775** **[M+2H]**	
***m*/*z***	**Relative Intensity**	***m*/*z***	**Relative Intensity**	***m*/*z***	**Relative Intensity**	***m*/*z***	**Relative Intensity**	***m*/*z***	**Relative Intensity**	***m*/*z***	**Relative Intensity**	***m*/*z***	**Relative Intensity**
58.07	0.74	70.07	65.23	70.07	100.00	58.07	0.76	70.07	2.45	58.07	27.46	84.08	8.41
70.07	3.38	86.10	21.66	86.10	44.91	70.07	1.26	72.08	1.04	70.07	50.18	86.10	12.23
72.08	2.46	116.07	12.41	137.05	27.41	72.08	0.74	84.08	1.76	72.08	45.56	110.07	4.69
81.03	40.01	141.10	8.06	155.08	44.56	77.01	1.49	86.10	1.65	84.04	19.65	120.08	5.07
82.04	1.13	152.06	100.00	183.15	20.06	81.03	0.76	97.03	3.07	84.08	34.24	126.06	5.00
84.04	1.40	169.10	15.10	187.07	6.82	84.08	0.98	125.03	1.82	86.10	100.00	129.10	14.21
84.08	2.28	183.15	26.43	197.13	6.14	86.10	1.94	129.10	1.48	88.04	12.81	141.10	12.70
86.10	5.32	197.13	8.24	203.14	8.74	95.02	4.73	139.05	1.99	110.07	40.86	147.11	4.39
95.02	1.21	211.14	28.46	211.14	15.70	97.03	0.49	155.04	2.09	129.10	19.66	169.10	12.91
102.06	0.74	214.15	10.25	256.13	10.45	112.05	0.76	167.04	0.97	155.08	25.91	175.12	18.64
110.07	0.92	229.15	12.39	268.17	7.70	117.04	0.88	180.09	100.00	181.06	68.76	226.15	4.24
112.05	100.00	240.13	7.80	284.12	63.19	129.10	0.74	181.09	1.01	242.04	14.60	246.16	34.96
113.05	2.51	308.10	8.00	300.16	7.19	136.06	2.11	181.09	4.18	256.09	29.04	268.10	11.23
120.08	1.25	324.07	12.19	369.21	15.05	168.07	2.57	191.04	3.66	285.14	18.03	286.11	100.00
127.05	1.19	326.21	17.99	397.21	39.58	199.01	3.99	209.06	6.76	286.11	19.53	287.12	4.57
129.10	1.67	566.26	13.11	482.30	6.13	244.07	2.55	213.02	1.75	322.07	18.01	301.15	4.33
207.08	4.40	567.26	11.19	486.26	17.44	268.10	16.28	227.07	6.58	324.06	13.05	445.73	5.51
268.10	0.69	567.29	9.16	510.29	8.89	269.11	0.61	245.08	0.90	326.09	31.72	529.27	5.27
286.11	2.08	567.35	7.19	599.34	45.88	286.11	100.00	286.11	4.04	356.22	21.41	529.30	12.87
323.06	1.57	568.27	25.51	600.34	9.91	287.12	4.65	439.07	1.40	373.24	71.91	531.29	7.68

**Table A8:** The MS2 peak lists of the uncharacterized metabolites detected specifically in *A. musiformis*.

***m*/*z* 347.644** **[M+2H]**		***m*/*z* 297.120** **[M+2H]**		***m*/*z* 514.287** **[M+H]**		***m*/*z* 597.360** **[M+H]**		***m*/*z* 580.015** **[M+H]**		***m*/*z* 593.241** **[M+H]**	
***m*/*z***	**Relative Intensity**	***m*/*z***	**Relative Intensity**	***m*/*z***	**Relative Intensity**	***m*/*z***	**Relative Intensity**	***m*/*z***	**Relative Intensity**	***m*/*z***	**Relative Intensity**
56.05	13.56	70.03	8.79	70.07	100.00	70.07	100.00	97.03	15.10	70.07	10.95
60.05	10.27	74.02	10.53	72.08	24.34	86.10	26.87	136.06	83.03	84.08	56.74
70.07	26.30	74.06	12.44	86.10	33.61	98.06	8.56	172.95	14.16	85.03	7.65
72.08	10.67	84.08	34.93	127.09	8.60	126.05	7.99	178.07	40.52	86.10	9.17
74.02	19.68	86.10	5.55	141.10	8.93	155.08	20.01	232.08	22.13	104.11	10.02
74.06	74.58	88.04	100.00	155.08	26.24	183.15	31.82	234.91	12.70	129.10	100.00
84.04	18.40	104.11	28.78	169.10	12.12	211.14	27.26	250.09	19.28	139.09	7.69
84.08	66.67	116.03	11.45	169.13	11.17	242.00	5.29	250.96	13.55	184.11	8.55
86.10	34.00	129.10	33.50	183.15	11.60	268.17	12.83	252.92	77.68	189.09	7.85
88.04	95.68	134.04	43.91	197.13	9.91	324.07	6.35	268.97	12.61	209.09	21.33
102.06	10.93	136.06	26.23	211.14	8.00	329.03	8.07	270.93	22.54	217.08	10.30
116.03	21.93	136.08	8.40	228.01	7.00	337.22	5.56	290.00	17.53	226.12	36.56
129.10	79.14	144.07	6.84	229.15	12.77	345.00	8.44	330.93	27.16	244.13	20.69
130.05	11.67	177.03	15.72	231.10	12.30	365.22	8.32	348.94	16.85	272.12	9.60
134.04	100.00	189.09	6.87	243.98	7.96	387.22	15.67	351.95	26.59	286.11	8.62
217.08	15.77	209.09	7.25	254.15	6.61	478.30	9.06	386.02	14.80	308.10	8.33
226.12	13.60	217.08	5.43	268.17	8.56	484.28	33.79	404.03	18.08	357.10	7.94
230.08	12.52	226.12	11.46	360.01	8.39	485.28	4.74	465.98	60.22	359.16	18.95
326.09	11.98	236.99	7.77	401.20	10.75	597.36	25.45	483.99	100.00	460.20	7.61
347.17	28.71	296.07	9.84	514.29	22.25	598.36	4.95	507.01	16.47	593.24	46.09
