# Supplementary material for: Nematode-Trapping Fungi Produce Diverse Metabolites during Predator–Prey Interaction
Source: Metabolites. 2020 Mar 20;10(3):117. doi: 10.3390/metabo10030117 (PMC7143726; doi:10.3390/metabo10030117)
Supplement: Supplementary file 1 [file metabolites-10-00117-s001.pdf]

Supplementary Materials
for
Nematode-Trapping Fungi Produce Diverse Metabolites During Predator-Prey Interaction

Ting-Hao Kuo[1,†], Ching-Ting Yang[2,†], Hsin-Yuan Chang[1], Yen-Ping Hsueh[2,*], Cheng-Chih Hsu[1,*]

1 Department of Chemistry, National Taiwan University, Taipei 10617, Taiwan

2 Institute of Molecular Biology, Academia Sinica, Taipei 115, Taiwan

* Correspondence: pinghsueh@gate.sinica.edu.tw (Y.-P.H.); ccrhsu@ntu.edu.tw (C.-C.H.)

† Contributed equally and should thus be considered as co-first authors.

Table of contents:

(i) Supplementary information of the metabolomic analyses of NTF

Table S1. List of the 100 *Arthrobotrys* strains isolated in Taiwan.

Table S2. List of the putatively characterized *Arthrobotrys musiformis* trap-associated peptides (AmTPs).

Table S3. List of the characterized desferriferrichrome analogues produced by *Arthrobotrys* fungi.

Table S4. List of the identified metabolites produced by *Arthrobotrys* fungi in the predatory stage.

Figure S1. Molecular networking of the aqueous metabolic extracts of the 100 *Arthrobotrys* strains during the fungi-nematode interaction.

Figure S2. Tandem mass spectrometric interrogation of the singly-charged Arthrobotrys musiformis trap-associated peptides (AmTPs).

Figure S3. Tandem mass spectral comparison between desferriferrichrome and its deoxygenated analogue.

Figure S4. Validation of the identified fungal metabolite - linoleyl alcohol.

Figure S5. Validation of the identified fungal metabolite –nonadecanamide.

Figure S6. Validation of the identified fungal metabolite – citicoline.

Figure S7. The abundance of the citicoline in each *Arthrobotrys* strain.

(ii) List of the uncharacterized metabolites particularly detected and enriched in the predatory stage.

Table S5. The predicted molecular formula of the uncharacterized metabolites enriched in the organic extracts in the predatory stage of NTF using SIRIUS 4.0. The list of the MS/MS spectral peaks is provided in Appendix A.

Table S6. Linking the uncharacterized (organic) metabolites with the sample information in public metabolomics datasets by MASST.

Figure S8. Comparison of the MS/MS spectra of the uncharacterized fungal metabolites in the organic extracts with those in the public metabolomics datasets extracted by MASST.

Table S7. The predicted molecular formula of the uncharacterized metabolites enriched in the aqueous extracts in the predatory stage of NTF using SIRIUS 4.0. The list of the MS/MS spectral peaks is provided in Appendix B.

Table S8. Linking the uncharacterized (aqueous) metabolites with the sample information in public metabolomics datasets by MASST.

Figure S9. Comparison of the MS/MS spectra of the uncharacterized fungal metabolites in the aqueous extracts with those in the public metabolomics datasets extracted by MASST.

(iii) Supplementary references

(i) Supplementary information of the metabolomic analyses of NTF

Table S1. List of the 100 *Arthrobotrys* strains isolated in Taiwan.

No.	Strain name	Species
**1**	TWF95	*A. oligospora*
**2**	TWF102	*A. oligospora*
**3**	TWF103	*A. oligospora*
**4**	TWF106	*A. oligospora*
**5**	TWF107	*A. oligospora*
**6**	TWF128	*A. oligospora*
**7**	TWF132	*A. oligospora*
**8**	TWF145	*A. oligospora*
**9**	TWF149	*A. oligospora*
**10**	TWF152	*A. oligospora*
**11**	TWF154	*A. oligospora*
**12**	TWF162	*A. oligospora*
**13**	TWF173	*A. oligospora*
**14**	TWF197	*A. oligospora*
**15**	TWF191	*A. oligospora*
**16**	TWF192	*A. oligospora*
**17**	TWF217	*A. oligospora*
**18**	TWF225	*A. oligospora*
**19**	TWF537	*A. oligospora*
**20**	TWF569	*A. oligospora*
**21**	TWF679	*A. oligospora*
**22**	TWF751	*A. oligospora*
**23**	TWF788	*A. oligospora*
**24**	TWF889	*A. oligospora*
**25**	TWF894	*A. oligospora*
**26**	TWF898	*A. oligospora*
**27**	TWF902	*A. oligospora*
**28**	TWF912	*A. oligospora*
**29**	TWF916	*A. oligospora*
**30**	TWF920	*A. oligospora*
**31**	TWF924	*A. oligospora*
**32**	TWF928	*A. oligospora*
**33**	TWF968	*A. oligospora*
**34**	TWF978	*A. oligospora*
**35**	TWF971	*A. oligospora*
**36**	TWF974	*A. oligospora*
**37**	TWF981	*A. oligospora*
**38**	TWF706	*A. oligospora*
**39**	TWF770	*A. oligospora*
**40**	TWF996	*A. oligospora*
**41**	ATCC24927	*A. oligospora*
**42**	TWF561	*A. oligospora*
**43**	TWF789	*A. oligospora*
**44**	TWF790	*A. oligospora*
**45**	TWF707	*A. oligospora*
**46**	TWF771	*A. oligospora*
**47**	TWF529	*A. oligospora*
**48**	TWF564	*A. oligospora*
**49**	TWF565	*A. oligospora*

**50**	TWF594	*A. oligospora*
**51**	TWF595	*A. oligospora*
**52**	TWF570	*A. oligospora*
**53**	TWF997	*A. oligospora*
**54**	TWF998	*A. oligospora*
**55**	TWF999	*A. oligospora*
**56**	TWF1000	*A. oligospora*
**57**	TWF703	*A. oligospora*
**58**	TWF704	*A. oligospora*
**59**	TWF705	*A. oligospora*
**60**	TWF752	*A. oligospora*
**61**	TWF753	*A. oligospora*
**62**	TWF754	*A. oligospora*
**63**	TWF538	*A. oligospora*
**64**	TWF539	*A. oligospora*
**65**	TWF156	*A. thaumasia*
**66**	TWF258	*A. thaumasia*
**67**	TWF678	*A. thaumasia*
**68**	TWF547	*A. thaumasia*
**69**	TWF566	*A. thaumasia*
**70**	TWF586	*A. thaumasia*
**71**	TWF588	*A. thaumasia*
**72**	TWF735	*A. thaumasia*
**73**	TWF291	*A. thaumasia*
**74**	TWF1005	*A. thaumasia*
**75**	TWF213	*A. thaumasia*
**76**	TWF233	*A. thaumasia*
**77**	TWF677	*A. thaumasia*
**78**	TWF287	*A. thaumasia*
**79**	TWF579	*A. thaumasia*
**80**	TWF741	*A. thaumasia*
**81**	TWF1006	*A. thaumasia*
**82**	TWF806	*A. thaumasia*
**83**	TWF105	*A. musiformis*
**84**	TWF140	*A. musiformis*
**85**	TWF166	*A. musiformis*
**86**	TWF556	*A. musiformis*
**87**	TWF481	*A. musiformis*
**88**	TWF571	*A. musiformis*
**89**	TWF697	*A. musiformis*
**90**	TWF791	*A. musiformis*
**91**	TWF906	*A. musiformis*
**92**	TWF784	*A. musiformis*
**93**	TWF161	*A. musiformis*
**94**	TWF175	*A. musiformis*
**95**	TWF563	*A. musiformis*
**96**	TWF482	*A. musiformis*
**97**	TWF700	*A. musiformis*
**98**	TWF778	*A. musiformis*
**99**	TWF780	*A. musiformis*
**100**	TWF907	*A. musiformis*

Table S2. List of the putatively characterized *Arthrobotrys musiformis* trap-associated peptides (AmTPs).

The peptides were reported in level 3 of metabolomic ID (i.e. putatively characterized compound classes based upon spectral similarity to known compounds of a chemical class) [1]. Common amino acids are abbreviated by single-letter nomenclature. The abbreviation for uncommon amino acids: DHIle, dehydroisoleucine; Abu, amino butyric acid.

***Note**: The amino acid sequences identified by the MS/MS fragments (in **Figure 3d** and **Figure S2**) are shown as bold and underlined letters; the ambiguous sequences/structures are shown as shaded letters and required further validation. The cyclic forms of the five AmTPs (AmTP-1, AmTP-2, … AmTP-5) remain to be validated with purified compounds or synthetic peptide standards.

***Arthrobotrys musiformis* trap-associated peptides (AmTPs)**	**Metabolomic ID level**	**MS/MS-proposed amino acid sequence**	**Predicted chemical formula**	**Detected ion**	**Exp. *m/z***	**Theo. *m/z***	**Mass error (ppm)**	**RT (min)**
AmTP-1	3	cyclic E-T-**A-R-A-I/L-S-I/L**-A-V	$C_{44}H_{77}N_{13}O_{14}$	$[M+2H]^{2+}$	506.7940	506.7929	2.17	5.85
AmTP-2	3	cyclic E-T-**A-R-A-I/L-S-I/L**-T-DHIle	$C_{46}H_{79}N_{13}O_{15}$	$[M+2H]^{2+}$	527.7989	527.7982	0.568	6.02
AmTP-3	3	cyclic E-T-**A-R-A-I/L-S-I/L-F**-(acetyl)L	$C_{53}H_{85}N_{13}O_{15}$	$[M+2H]^{2+}$	572.8214	572.8217	0.523	6.75
AmTP-4	3	cyclic E-T-**A-R-A-I/L-S-T-A-I/L**-S	$C_{46}H_{80}N_{14}O_{17}$	$[M+2H]^{2+}$	551.3005	551.2986	1.63	7.15
AmTP-5	3	cyclic E-T-**A-R-A-I/L-S-I/L-F**-L	$C_{51}H_{83}N_{13}O_{14}$	$[M+2H]^{2+}$	551.8172	551.8164	1.45	6.01
AmTP-6	3	T-DHIle-**I/L-S-I/L**-($C_6H_{14}O_2N$)	$C_{31}H_{59}N_7O_9$	$[M+H]^+$	674.4443	674.4447	0.445	7.12
AmTP-7	3	T-DHIle-**I/L-S-I/L**-A	$C_{28}H_{50}N_6O_9$	$[M+H]^+$	615.3717	615.3712	0.813	6.62
AmTP-8	3	T-DHIle-**I/L-S-I/L**-Abu	$C_{29}H_{52}N_6O_9$	$[M+H]^+$	629.3864	629.3869	0.794	7.03
AmTP-9	3	I/L-G-**I/L-S-I/L**-Abu	$C_{27}H_{50}N_6O_8$	$[M+H]^+$	587.3766	587.3763	0.511	6.55
AmTP-10	3	I/L-G-**I/L-S-I/L**-A	$C_{26}H_{48}N_6O_8$	$[M+H]^+$	573.3614	573.3606	1.40	6.18
		Note:<br>(1) **unambiguous sequences** identified by MS/MS fragments (see **Figure 3d** and **Figure S2**) are bold and underlined.<br>(2) ambiguous sequences are shaded.<br>(3) Both the ambiguous sequences and the cyclic forms remain to be validated with purified compounds or synthetic peptide standards.						

Table S3. List of the characterized desferriferrichrome analogues produced by *Arthrobotrys* fungi.

Identification of desferriferrichrome was validated with chemical standard and reported in level 1 of metabolomic ID (i.e. a minimum of two independent and orthogonal data relative to an authentic compound analyzed under identical experimental conditions). The characterized analogues of desferriferrichrome were reported in level 3 of metabolomic ID (i.e. putatively characterized compound classes based upon by spectral similarity to known compounds of a chemical class).

Siderophore	Metabolomic ID level	Chemical formula	Exact mass (Da)	Chemical structure	Detected ion	Exp. *m/z*	Theo. *m/z*	Mass error (ppm)	RT (min)
Desferriferrichrome	1	$C_{27}H_{45}N_9O_{12}$	687.3188	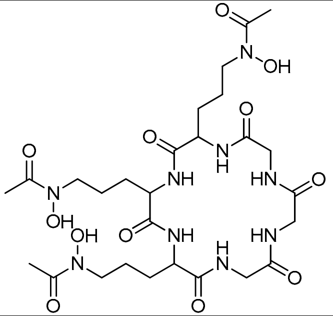	$[M+H]^+$	688.3256	688.3260	0.581	3.20
Deoxygenated desferriferrichrome	3	$C_{27}H_{45}N_9O_{11}$	671.3239	(MS/MS proposed analogue)	$[M+H]^+$	672.3312	672.3311	0.149	2.61
Deacetylated desferriferrichrome	3	$C_{25}H_{41}N_9O_{11}$	643.2926	(MS/MS proposed analogue)	$[M+H]^+$	644.2992	644.2998	0.931	2.48
Acetylated desferriferrichrome	3	$C_{29}H_{47}N_9O_{13}$	729.3293	(MS/MS proposed analogue)	$[M+2H]^{2+}$	365.6718	365.6720	0.547	5.29

Table S4. List of the identified metabolites produced by *Arthrobotrys* fungi in the predatory stage.

Identification of these metabolites was validated with chemical standards and reported in level 1 of metabolomic ID (i.e. a minimum of two independent and orthogonal data relative to an authentic compound analyzed under identical experimental conditions).

Metabolite	Metabolomic ID level	Chemical formula	Chemical structure	Detected ion	Exp. m/z	Theo. m/z	Mass error (ppm)	RT (min)
Linoleic alcohol	1	$C_{18}H_{34}O$	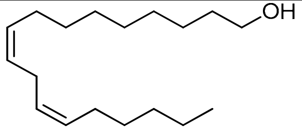	$[M+H]^+$	267.2679	267.2682	1.12	11.71
Nonadecanamide	1	$C_{19}H_{39}NO$	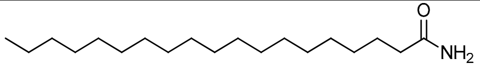	$[M+H]^+$	298.3099	298.3104	1.69	8.02
Citicoline	1	$C_{14}H_{27}N_4O_{11}P_2^+$	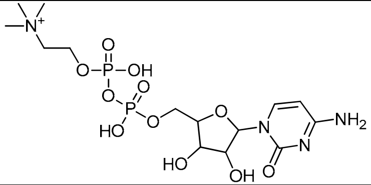	M^+	489.1143	489.1146	0.613	7.07

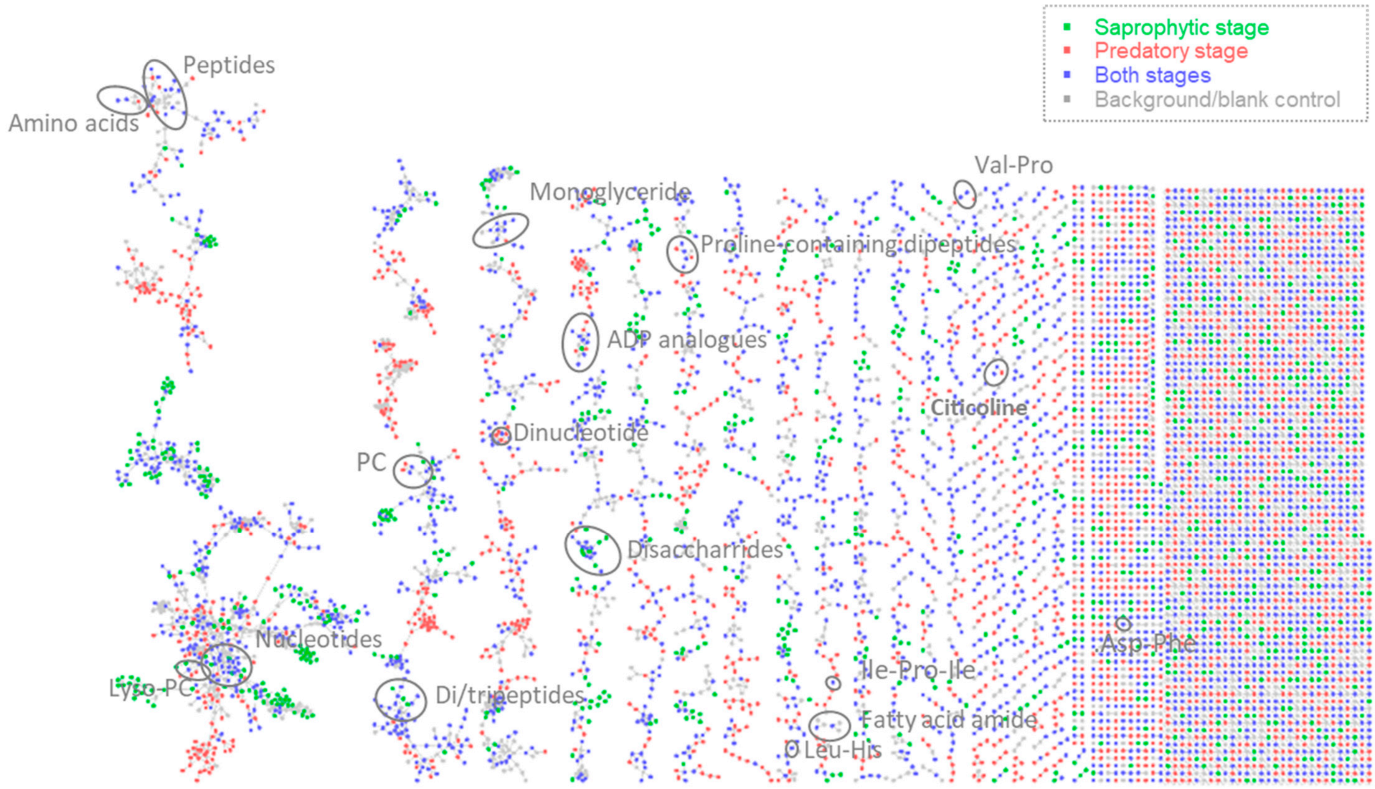

Figure S1. Molecular networking of the aqueous metabolite extracts of the 100 *Arthrobotrys* strains during the fungi-nematode interaction. The putatively annotated metabolites by searching against the GNPS database are highlighted.

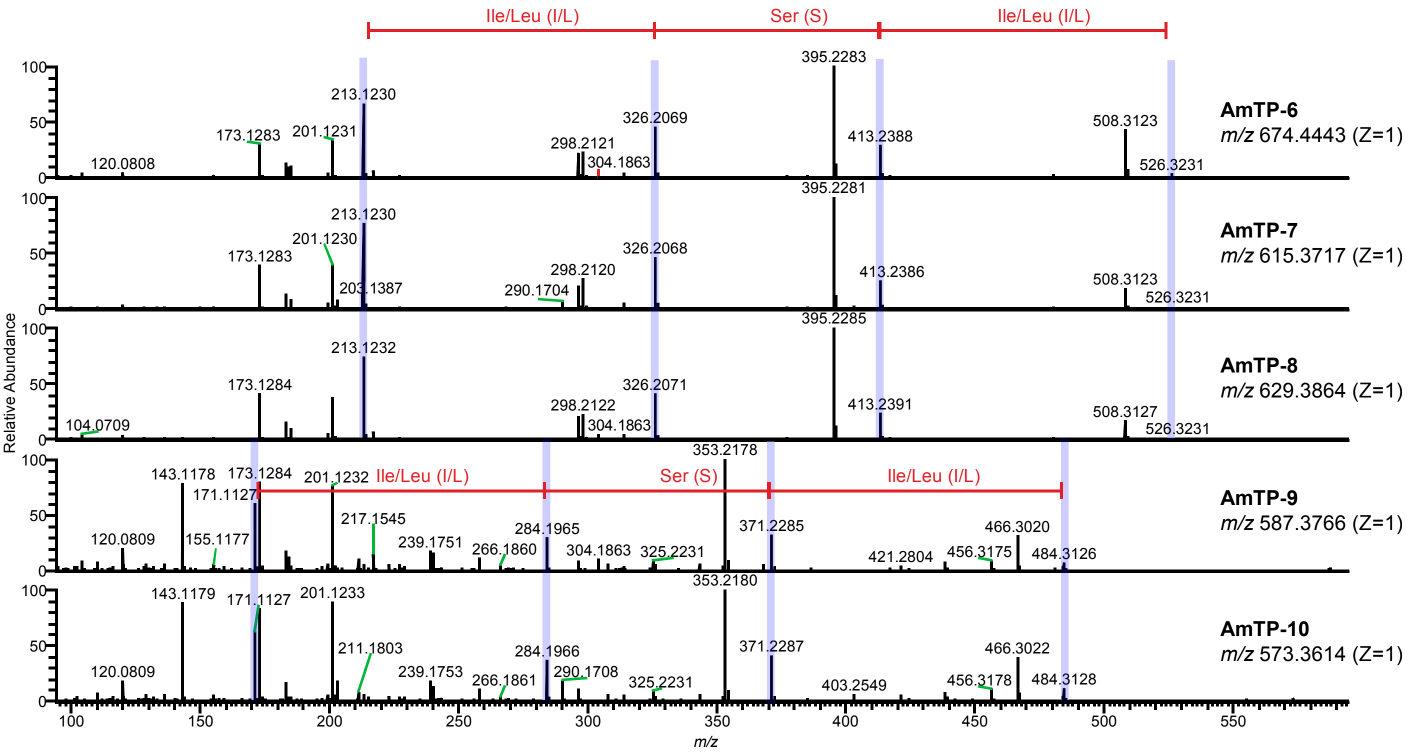

Figure S2. Tandem mass spectrometric interrogation of the singly-charged *Arthrobotrys musiformis* trap-associated peptides (AmTPs).

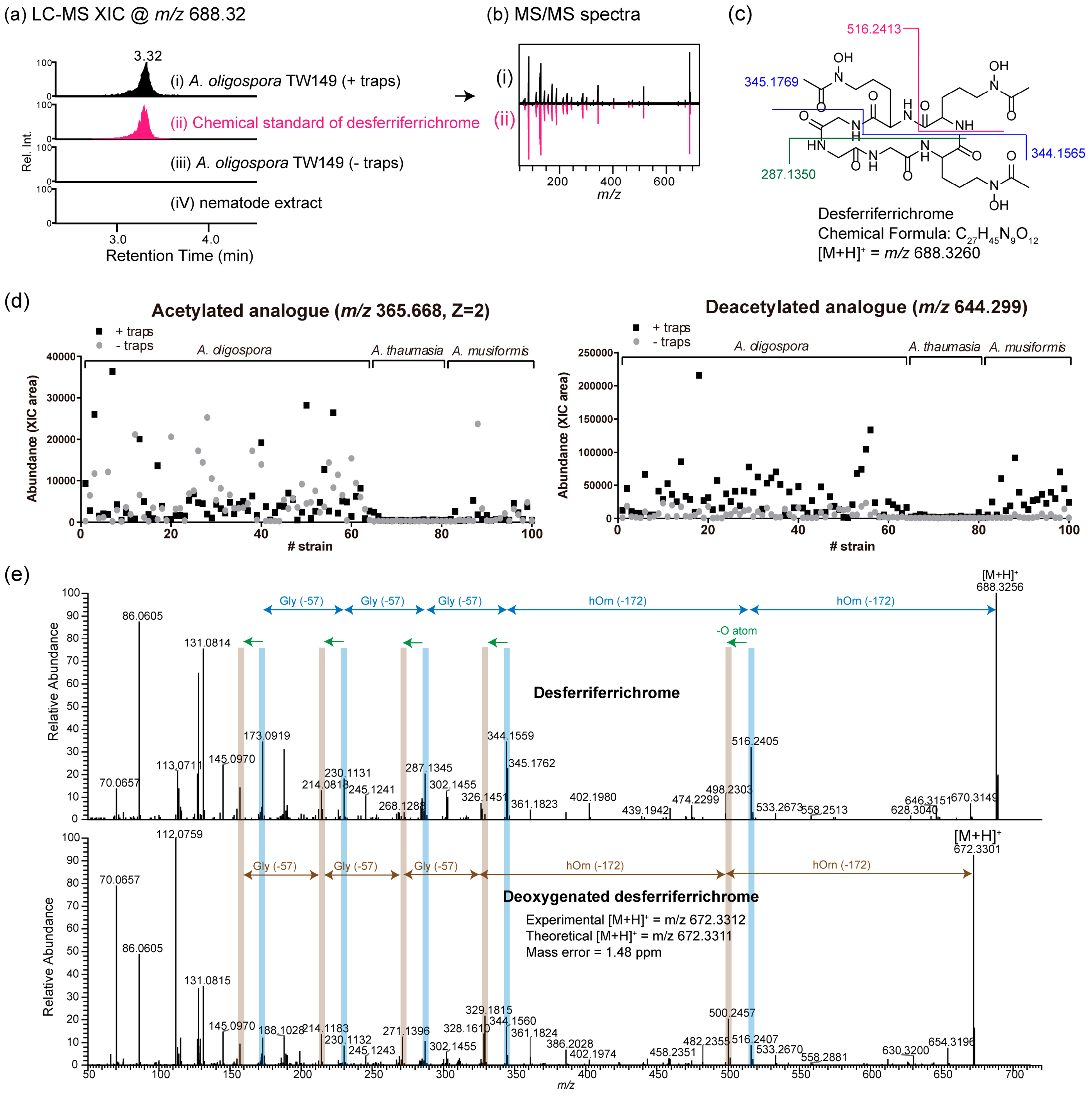

Figure S3. Tandem mass spectral comparison between desferriferrichrome and its deoxygenated analogue.

(a) The extracted ion chromatogram (XIC) of desferriferrichrome (*m/z* 688.32) in the samples of (i) the extract of *A. oligospora* TW149 in the predatory stage (ii) the chemical standard (iii) the extract of *A. oligospora* TW149 in the saprophytic stage (iv) the extract of *C. elegans*. (b) Comparison of the MS/MS spectra of desferriferrichrome obtained in the extract of *A. oligospora* TW149 in the predatory stage and the chemical standard. (c) The proposed fragmentation scheme of desferriferrichrome. (d) The abundance of acetylated desferriferrichrome (left panel) and deacetylated desferriferrichrome (right panel) in each *Arthrobotrys* strain before and after forming the traps. Abundance was estimated by LC-MS peak area. (e) Tandem mass spectral comparison between desferriferrichrome and its deoxygenated analogue. Fragments indicating difference of an oxygen atom, hydroxamating ornithine, or glycine monomers are highlighted.

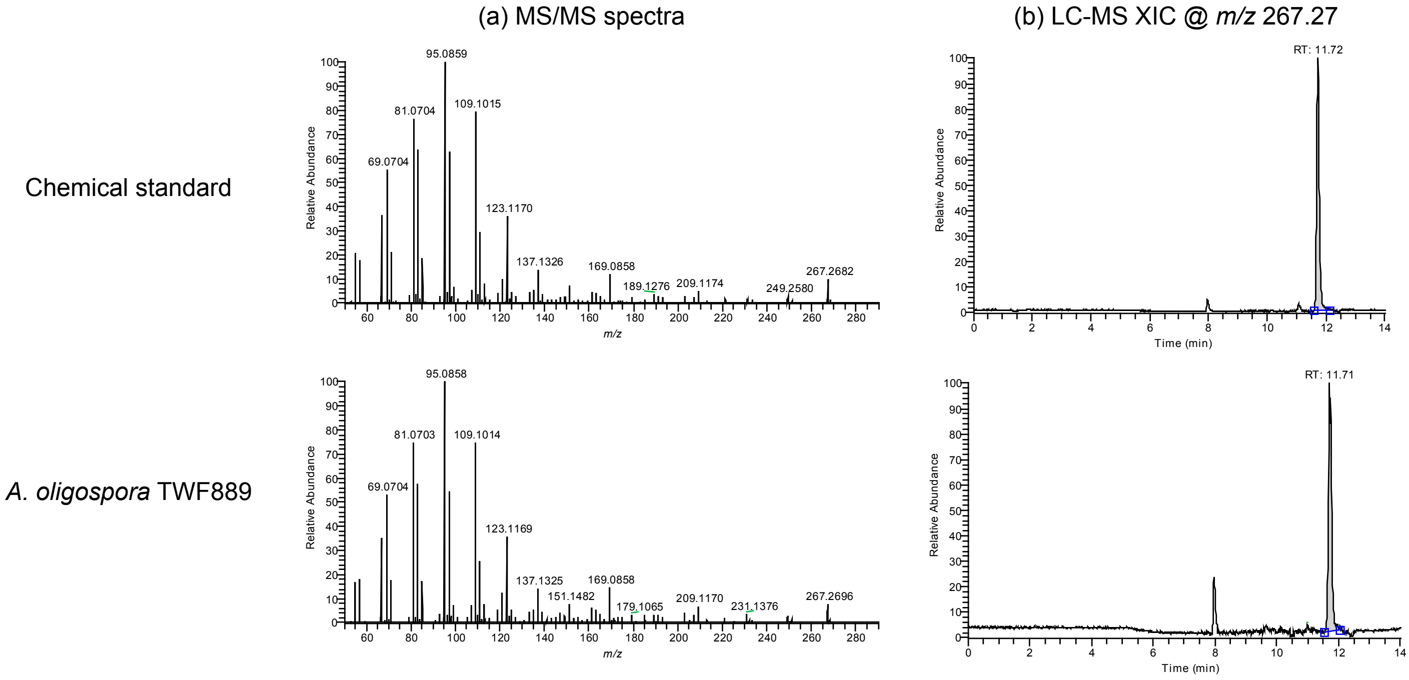

Figure S4. Validation of the identified fungal metabolite - linoleyl alcohol.

The high-resolution MS/MS spectra and extracted ion chromatograms (XIC) obtained from the chemical standard of linoleyl alcohol were shown in (a) and (b), respectively.

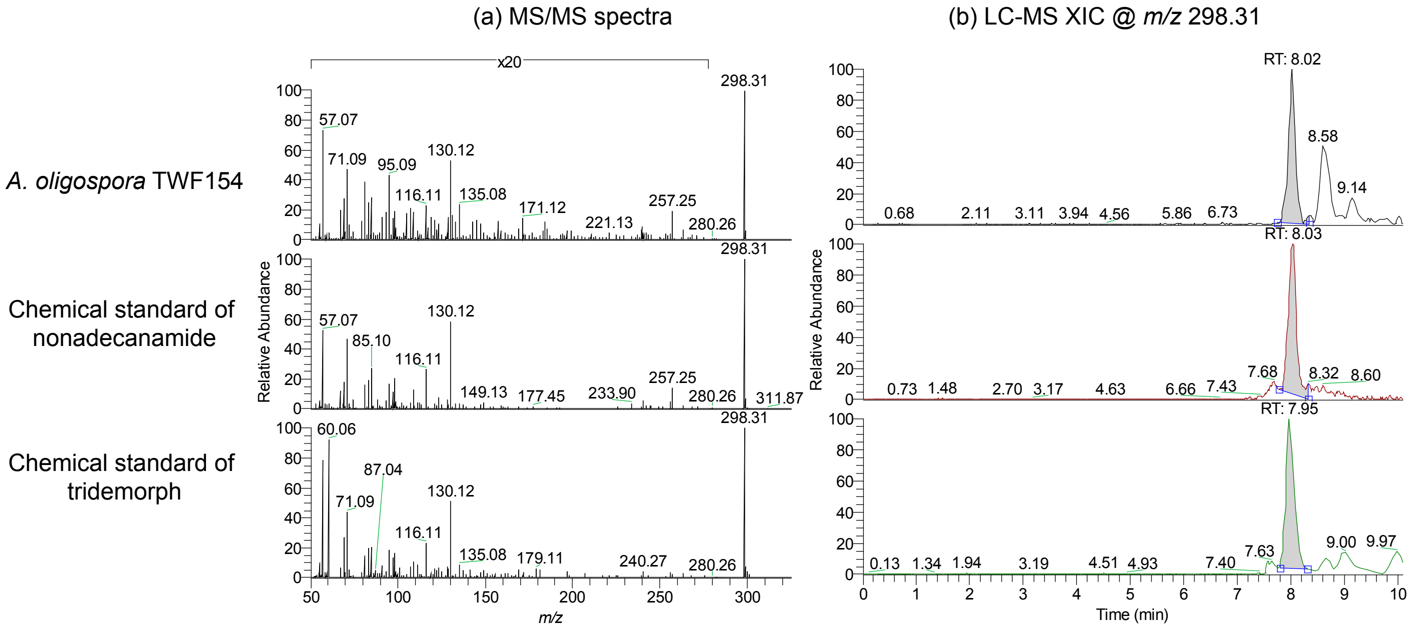

Figure S5. Validation of the identified fungal metabolite –nonadecanamide.

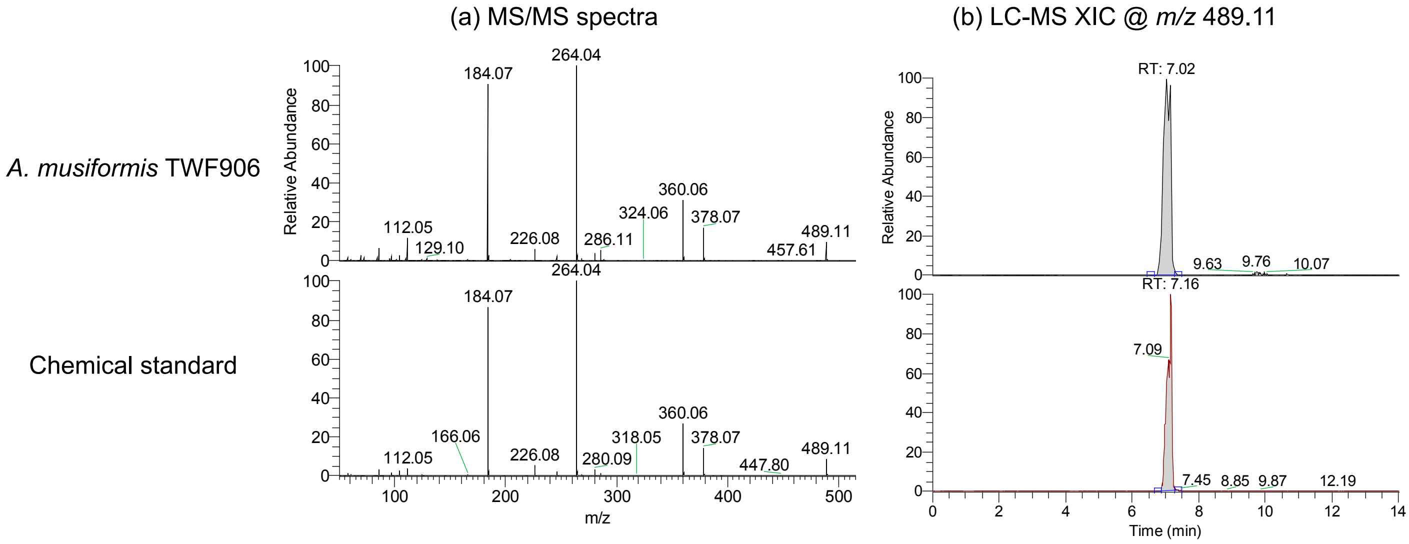

Figure S6. Validation of the identified fungal metabolite – citicoline.

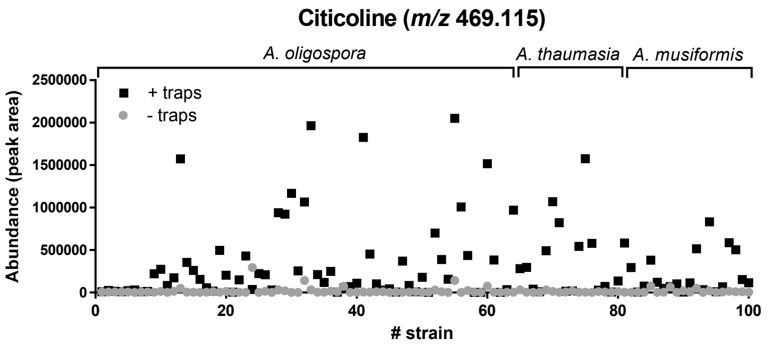

Figure S7. The abundance of the citicoline in each *Arthrobotrys* strain. Abundance was estimated by LC-MS peak area.

(ii) List of the uncharacterized metabolites particularly detected and enriched in the predatory stage.

Table S5. The predicted molecular formula of the uncharacterized metabolites enriched in the organic extracts in the predatory stage of NTF using SIRIUS 4.0 [2]. The list of the MS/MS spectral peaks is provided in **Appendix A**.

a. The predicted molecular formula of the metabolites detected commonly in *A. oligospora, A. thaumasia, and A. musiformis*.

m/z	Adduct	Predicted Molecular Formula (Score %)
454.229	$[M+H]^+$	$C_{20}H_{31}N_5O_7$ (98.71 %)
376.144	$[M+H]^+$	$C_{20}H_{17}N_5O_3$ (75.43 %)
519.193	$[M+H]^+$	$C_{16}H_{26}N_{10}O_{10}$ (99.69 %)
462.208	$[M+H]^+$	$C_{15}H_{34}N_4O_{10}P$ (92.74 %)
590.230	$[M+H]^+$	$C_{20}H_{27}N_{15}O_7$ (67.91 %)
		$C_{23}H_{35}N_5O_{13}$ (6.71 %)
726.281	$[M+H]^+$	$C_{27}H_{39}N_{11}O_{13}$ (32.88 %)
		$C_{43}H_{39}N_3O_8$ (17.34 %)
293.276	$[M+H]^+$	$C_{11}H_{32}N_8O$ (100.00 %)
317.191	$[M+H]^+$	$C_8H_{20}N_{12}O_2$ (94.59%)
372.311	$[M+H]^+$	$C_{19}H_{39}N_4O_3$ (100.00 %)
768.539	$[M+H]^+$	$C_{46}H_{73}NO_8$ (14.38 %)
376.145	$[M+2H]^{2+}$	$C_{13}H_{21}N_5O_8$ (81.25 %)
529.409	$[M+H]^+$	$C_{26}H_{52}N_6O_5$ (97.79 %)
184.606	$[M+H]^+$	N/A

b. The predicted molecular formula of the metabolites detected specifically in *A. oligospora*.

m/z	Adduct	Predicted Molecular Formula (Score %)
209.128	$[M+H]^+$	$C_{11}H_{16}N_2O_2$ (99.22 %)

c. The predicted molecular formula of the metabolites detected specifically in *A. thaumasia*.

m/z	Adduct	Predicted Molecular Formula (Score %)
216.042	$[M+H]^+$	$C_8H_{10}NO_2PS$ (100.00 %)
259.992	$[M+H]^+$	$C_5H_{13}NO_3P_4$

		(99.51 %)
338.171	$[M+H]^+$	$C_{10}H_{24}N_7O_4P$ (85.87 %)
339.249	$[M+H]^+$	$C_{16}H_{30}N_6O_2$ (44.71 %)
368.182	$[M+H]^+$	$C_{18}H_{21}N_7O_2$ (63.30 %)
423.005	$[M+H]^+$	$C_{16}H_{13}N_2O_6P_3$ (36.19 %)
443.287	$[M+H]^+$	$C_{22}H_{34}N_8O_2$ (48.66 %)
		$C_{21}H_{38}N_4O_6$ (45.35 %)
344.662	$[M+H]^+$	N/A

d. The predicted molecular formula of the metabolites detected specifically in *A. musiformis*.

m/z	Adduct	Predicted Molecular Formula (Score %)
250.003	$[M+H]^+$	$C_7H_9NO_5P_2$ (28.64 %)
		$C_8H_{11}NO_2S_3$ (28.64 %)
261.989	$[M+H]^+$	$C_5H_{13}NO_3P_2S_2$ (25.47 %)
271.202	$[M+H]^+$	$C_{14}H_{26}N_2O_3$ (100.00 %)
323.200	$[M+H]^+$	$C_{22}H_{26}O_2$ (97.44 %)
354.239	$[M+H]^+$	$C_{18}H_{31}N_3O_4$ (93.71 %)
361.217	$[M+H]^+$	$C_{10}H_{24}N_{12}O_3$ (70.34 %)
373.244	$[M+H]^+$	$C_{11}H_{33}N_8O_4P$ (48.62 %)
390.099	$[M+H]^+$	$C_{10}H_{19}N_3O_{13}$ (48.62 %)
443.287	$[M+H]^+$	$C_{22}H_{34}N_8O_2$ (89.05 %)
519.265	$[M+H]^+$	$C_{16}H_{39}N_8O_9P$ (44.34 %)
550.446	$[M+2H]^{2+}$	$C_{29}H_{55}N_7O_3$ (84.36 %)
945.327	$[M+H]^+$	$C_{50}H_{44}N_{10}O_{10}$ (20.53 %)
		$C_{45}H_{44}N_{12}O_{12}$ (20.53 %)

Table S6. Linking the uncharacterized (organic) metabolites with the sample information in public metabolomics datasets by MASST [3]. The table includes the successful MASST searching results of the uncharacterized metabolites listed in **Table S5.** The corresponding MS/MS spectra are provided in **Figure S8**.

**Target *m/z***	**# Match spectra**	**Cosine (matched score, %)**	**∆MZ (Da)**	**Organisms**	**MS/MS Spectra (see below Figure S8)**
462.208	1	75	1.84	*Zea mays* subsp. (mays)	**a**
590.230	2	75	0	environmental samples <*Bacillariophyta*>	**b**
590.230		73	0	Coral	**c**
372.311	1	70	1.58	C57BL\/6N Mice	**d**
259.992	1	84	0	environmental samples <*Bacillariophyta*>	**e**
338.171	3	70	1.26	*Homo sapiens*	**f**
		70	1.98	*Phaeomoniella chlamydospora; Phaeoacremonium aleophilum; Eutypa lata*	**g**
		70	1.26	*Homo sapiens*	**h**
339.249	1	80	1.17	*Solanum lycopersicum* (tomato)	**i**
368.182	1	74	0	*Mus musculus*	**j**
443.287	1	76	1.02	*Mus musculus*	k
344.662	2	78	0.03	*Zea mays* (Corn)	**l**
344.662		70	0	Food metagenome	**m**
261.989	1	72	0.18	environmental samples <*Bacillariophyta*>	**n**
271.202	2	74	0.91	*Rattus*	**o**
		71	0.79	*Mus musculus*	**p**
443.287	1	74	0.98	*Mus musculus*	**q**

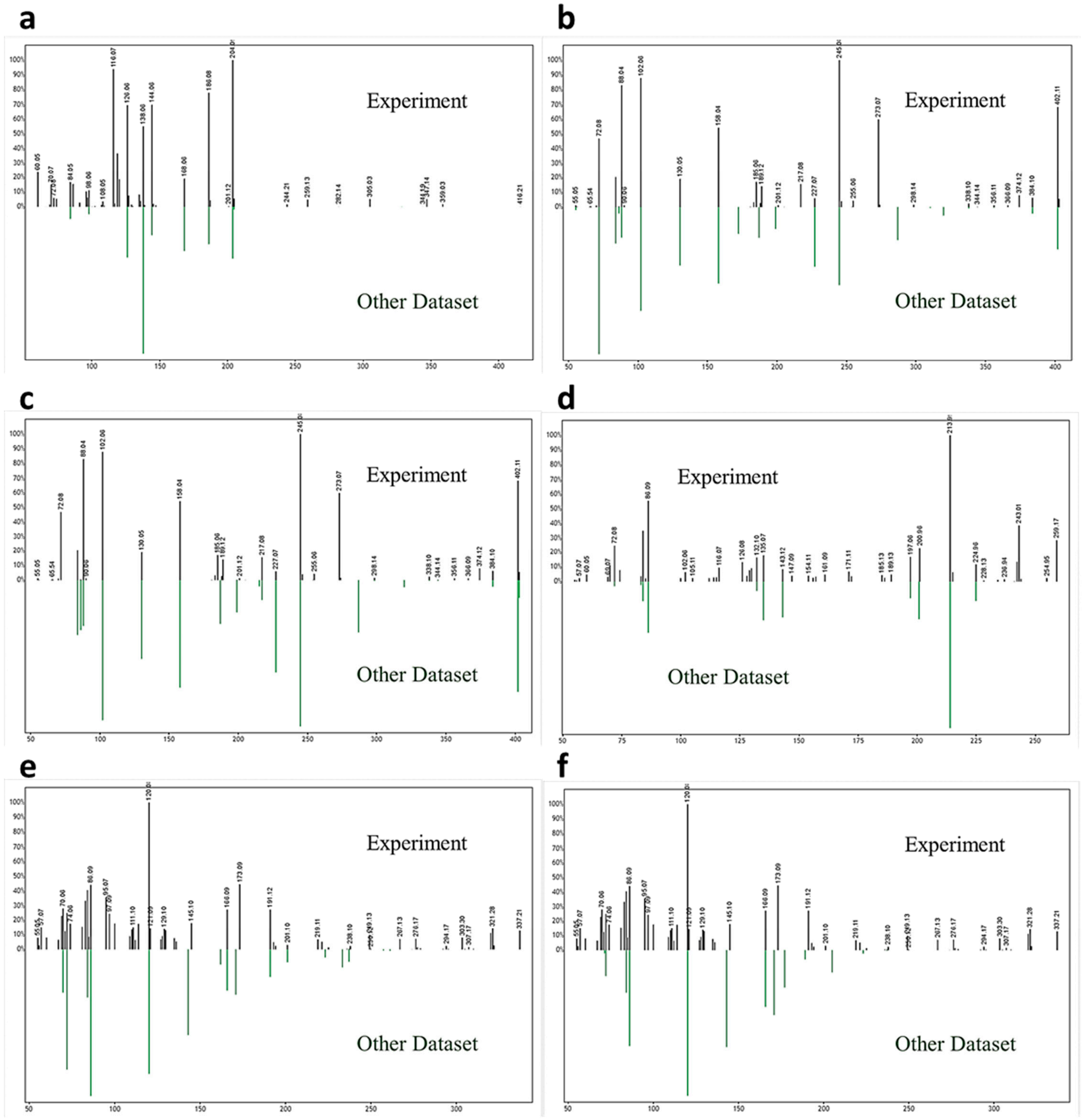

Figure S8. Comparison of the MS/MS spectra of the uncharacterized fungal metabolites in the organic extracts with those in the public metabolomics datasets extracted by MASST. Metabolites in sub-figures refer to **Table S6**. (continuing)

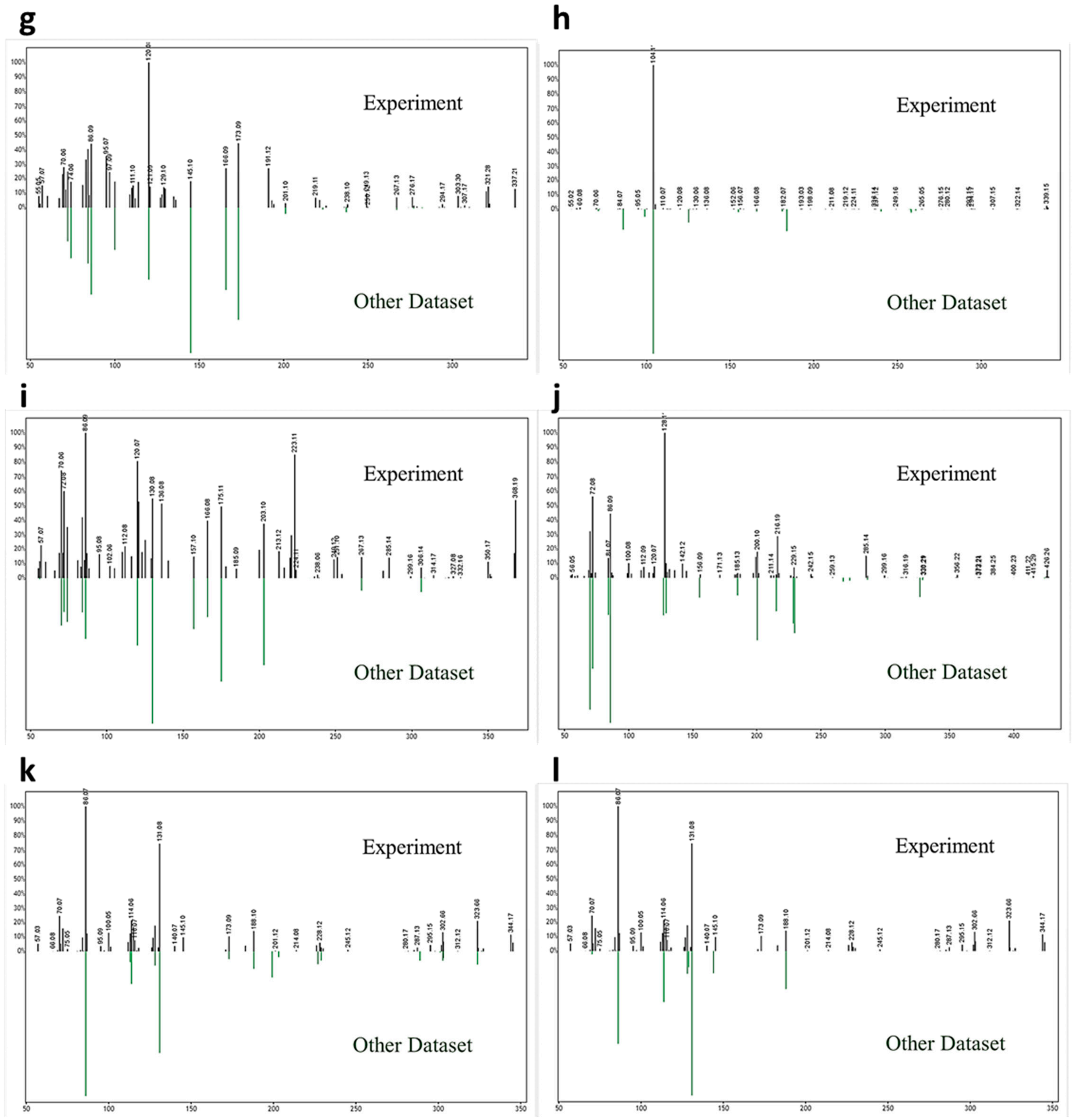

Figure S8. Comparison of the MS/MS spectra of the uncharacterized fungal metabolites in the organic extracts with those in the public metabolomics datasets extracted by MASST. Metabolites of sub-figures refer to **Table S6**. (continuing)

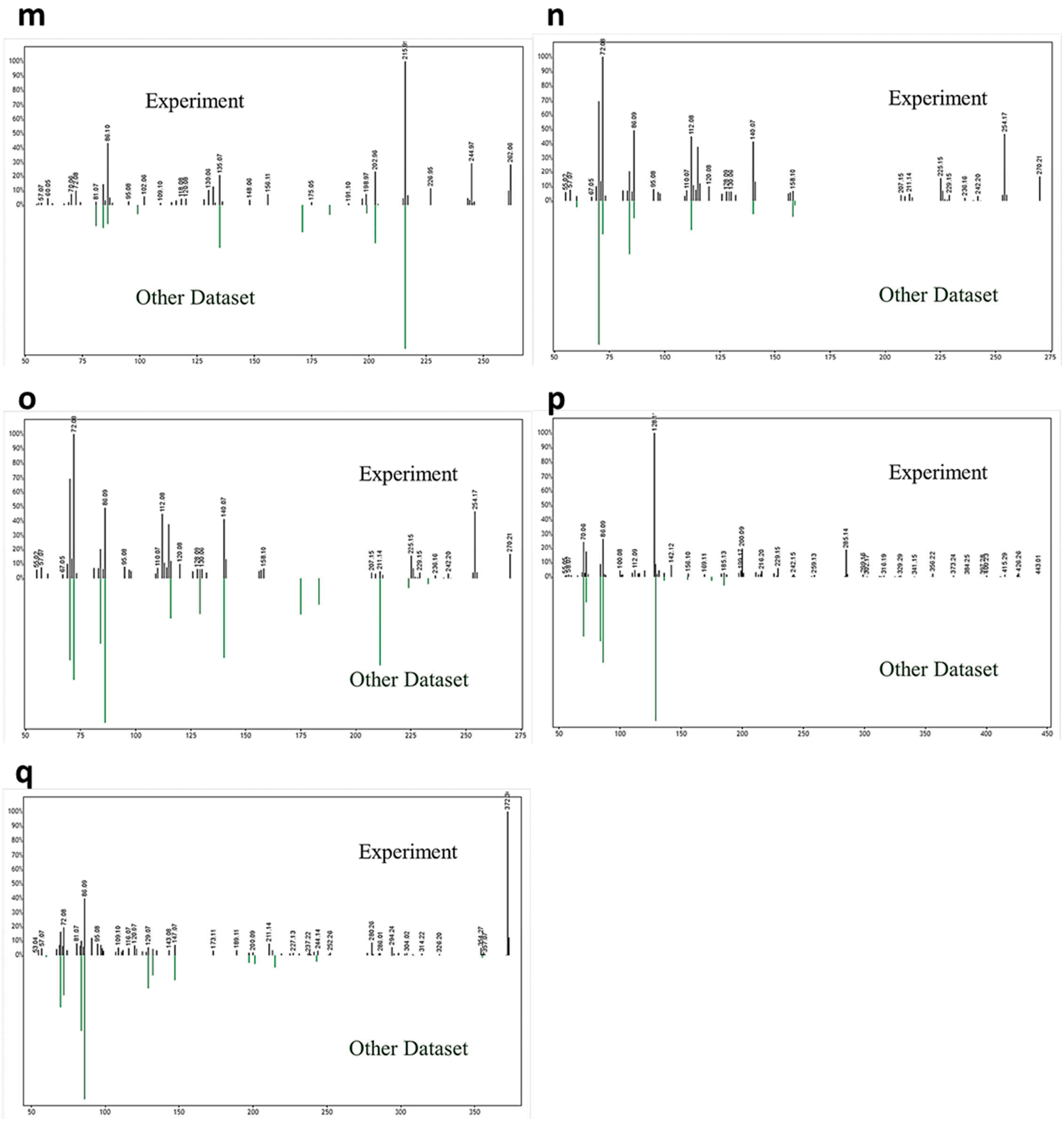

Figure S8. Comparison of the MS/MS spectra of the uncharacterized fungal metabolites in the organic extracts with those in the public metabolomics datasets extracted by MASST. Metabolites of sub-figures refer to **Table S6**.

Table S7. The predicted molecular formula of the uncharacterized metabolites enriched in the aqueous extracts of NTF in the predatory stage using SIRIUS 4.0. The list of the MS/MS spectral peaks is provided in **Appendix B**.

a. The predicted molecular formula of the metabolites detected commonly in *A. oligospora, A. thaumasia, and A. musiformis*.

m/z	Adduct	Predicted Molecular Formula (Score %)
413.276	$[M+H]^+$	$C_{11}H_{34}N_{13}O_2S$ (41.17 %)
750.558	$[M+H]^+$	$C_{37}H_{75}N_5O_{10}$ (31.59 %)
		$C_{38}H_{71}N_9O_6$ (29.67 %)
314.207	$[M+H]^+$	$C_{15}H_{27}N_3O_4$ (99.84 %)
455.189	$[M+H]^+$	$C_{19}H_{22}N_{10}O_4$ (58.78 %)
		$C_{18}H_{26}N_6O_8$ (17.55 %)
484.318	$[M+H]^+$	$C_{17}H_{41}N_9O_7$ (85.87 %)
498.328	$[M+H]^+$	$C_{20}H_{39}N_{11}O_4$ (50.00 %)
		$C_{25}H_{39}N_9O_2$ (49.99 %)
528.302	$[M+H]^+$	$C_{24}H_{41}N_5O_8$ (91.15 %)
565.167	$[M+H]^+$	$C_{13}H_{30}N_{10}O_{11}P_2$ (46.86 %)
		$C_{17}H_{34}N_4O_{13}P_2$ (8.26 %)
145.170	$[M+H]^+$	$C_8H_{20}N_2$ (100.00 %)
422.065	$[M+H]^+$	$C_{10}H_7N_{13}O_7$ (34.11%)
		$C_{25}H_{11}NO_6$ (30.01%)
356.213	$[M+H]^+$	$C_{24}H_{25}N_3$ (95.32%)
418.167	$[M+H]^+$	$C_{14}H_{23}N_7O_8$ (96.88 %)
647.193	$[M+H]^+$	$C_{27}H_{30}N_6O_{13}$ (21.28 %)
		$C_{32}H_{31}N_4O_9P$ (4.26 %)

b. The predicted molecular formula of the metabolites detected specifically in *A. oligospora*.

m/z	**Adduct**	**Predicted Molecular Formula (Score %)**
485.610	$[M+2H]^+$	$C_{35}H_{37}N_{15}O_{15}P_2$<br>$C_{37}H_{32}N_{17}O_{14}P$<br>$C_{24}H_{38}N_{21}O_{16}P_3$<br>$C_{32}H_{32}N_{19}O_{16}P$<br>(12.51 %)

461.605	$[M+2H]^+$	N/A
423.172	$[M+H]^+$	$C_{12}H_{25}N_9O_6P$ (62.45 %)
227.033	$[M+H]^+$	$C_8H_6N_2O_6$ (99.53 %)
299.543	$[M+2H]^+$	$C_{19}H_{17}N_{11}O_{10}$ (45.79 %)
557.150	$[M+H]^+$	$C_{20}H_{21}N_{12}O_6P$ (96.72 %)

c. The predicted molecular formula of the metabolites detected specifically in *A. thaumasia*.

m/z	**Adduct**	**Predicted Molecular Formula (Score %)**
228.050	$[M+H]^+$	$C_9H_9NO_6$ (99.91 %)
364.186	$[M+H]^+$	$C_{12}H_{26}N_7O_4P$ (99.72 %)
425.214	$[M+H]^+$	$C_{16}H_{33}N_4O_7P$ (42.89 %)
		$C_{17}H_{32}N_2O_{10}$ (35.00 %)
		$C_{18}H_{28}N_6O_6$ (17.56 %)
439.291	$[M+H]^+$	$C_{16}H_{39}N_8O_4P$ (64.52 %)
		$C_{22}H_{38}N_4O_5$ (35.28 %)
470.297	$[M+H]^+$	$C_{23}H_{35}N_9O_2$ (74.73 %)
512.344	$[M+H]^+$	$C_{21}H_{41}N_{11}O_4$ (99.97 %)
524.198	$[M+H]^+$	$C_{16}H_{30}N_9O_9P$ (36.79 %)
528.339	$[M+H]^+$	$C_{25}H_{45}N_5O_7$ (62.27 %)
		$C_{26}H_{41}N_9O_3$ (13.19 %)
532.144	$[M+H]^+$	$C_{21}H_{21}N_7O_{10}$ (58.71 %)
567.350	$[M+H]^+$	$C_{21}H_{47}N_{10}O_6P$ (45.80 %)
599.340	$[M+H]^+$	$C_{23}H_{42}N_{12}O_7$ (44.25 %)
		$C_{28}H_{42}N_{10}O_5$ (27.20 %)
617.182	$[M+H]^+$	$C_{22}H_{24}N_{12}O_{10}$ $C_{21}H_{28}N_8O_{14}$ (35.35 %)
618.155	$[M+H]^+$	$C_{17}H_{24}N_{13}O_{11}P$ (33.80 %)
		$C_{27}H_{27}N_3O_{14}$ (10.04 %)
347.653	$[M+2H]^+$	$C_{33}H_{35}N_{13}O_5$ (35.26 %)
		$C_{30}H_{39}N_{13}O_5S$ (7.05 %)

529.775	$[M+2H]^+$	N/A

d. The predicted molecular formula of the metabolites detected specifically in *A. musiformis*.

m/z	**Adduct**	**Predicted Molecular Formula (Score %)**
347.644	$[M+2H]^+$	$C_{23}H_{44}N_{13}O_6PS_2$ $C_{27}H_{50}N_7O_6P_3S$ (29.96 %)
297.120	$[M+2H]^+$	$C_{27}H_{36}N_4O_9S$ (33.33 %)
		$C_{28}H_{32}N_8O_5S$ (33.33 %)
514.287	$[M+H]^+$	$C_{28}H_{39}N_3O_6$ (46.85 %)
597.360	$[M+H]^+$	$C_{28}H_{48}N_6O_8$ (46.85 %)
580.015	$[M+H]^+$	$C_{13}H_{14}N_{10}O_{11}P_3$ (54.91 %)
		$C_{14}H_{20}N_3O_{16}P_3$ (34.46 %)
593.241	$[M+H]^+$	$C_{21}H_{33}N_{14}O_3PS$ (34.49 %)
		$C_{19}H_{38}N_{12}O_4P_2S$ (32.10 %)

Table S8. Linking the uncharacterized (aqueous) metabolites with the sample information in public metabolomics datasets by MASST. The table includes the successful MASST searching results of the uncharacterized metabolites listed in **Table S7.** The corresponding MS/MS spectra are provided in **Figure S9**.

**Target *m/z***	**Number of matched spectra**	**Cosine**	**ΔMZ (Da)**	**Sample information**	**MS/MS spectra (see Figure S9)**
413.276	4	0.79	0.07	*Homo sapiens* (Stool sample)	**a**
		0.78	0	mouse stool sample	**b**
		0.77	1.94	Environmental samples *<Bacillariophyta>*	**c**
		0.76	0	*Salinispora arenicola; Salinispora pacifica; Salinispora tropica*	**d**
750.558	3	0.87	0.01	*Solanum lycopersicum*	**e**
		0.86	0.01	*Homo sapiens* (Stool sample)	**f**
		0.84	0	*Ovis aries (*Sheep faeces)	**g**
314.207	1	0.85	0	*Mus musculus* fecal sample	**h**
484.318	1	0.74	0	*Mus musculus* fecal sample	**i**
498.328	2	0.72	0	*Mus musculus* fecal sample	**j**
		0.71	0	*Solanum lycopersicum*	**k**
565.166	3	0.94	0	*Mus musculus* fecal sample	**l**
		0.92	0.03	Environmental samples *<Bacillariophyta>*	**m**
		0.71	0.01	*Acropora yongei*	**n**
485.61	1	0.7	1.57	*Mus musculus* urogenital tract organs	**o**
557.15	1	0.71	0	*Mus musculus* fecal sample	**p**
228.050	1	0.74	1.04	*Streptomyces* and *Pseudomonas* species	**q**
364.186	3	0.84	1.98	*Phaeomoniella chlamydospora; Phaeoacremonium aleophilum; Eutypa lata*	**r**
		0.84	1.62	Samples of cotton leaves, fall armyworm midgut content and faeces.	**s**
		0.83	0	*Mus musculus* fecal sample	**t**
425.214	2	0.80	0.06	*Salinispora arenicola; Salinispora pacifica; Salinispora tropica*	**u**
		0.73	0.22	*Mus musculus* gut sample	**v**
439.291	6	0.91	0.01	food fermentation metagenome (Cheese culture)	**w**
		0.90	0	*Streptomyces sp.*	**x**
		0.89	0	*Pseudomonas aeruginosa; Arabidopsis thaliana*	**y**
		0.88	0.14	*Salinispora pacifica; Salinispora arenicola; Salinispora tropica*	**z**
		0.86	0.01	*Phaeomoniella chlamydospora; Phaeoacremonium aleophilum; Eutypa lata*	**aa**
		0.86	0	*Streptomyces* and *Pseudomonas* species	**ab**
470.297	1	0.71	0.19	*Mus musculus* fecal sample	**ac**
532.144	4	0.89	0	*Mus musculus* fecal sample	**ad**
		0.88	0	Environmental samples *<Bacillariophyta>*	**ae**

		0.89	0	*Homo sapiens* (from CF patients treated with *tobramycin)*	**af**
		0.72	0	*Pseudomonas aeruginosa*	**ag**
599.340	2	0.76	0.12	*Mus musculus* gut sample	**ah**
		0.75	0	food metagenome	**ai**

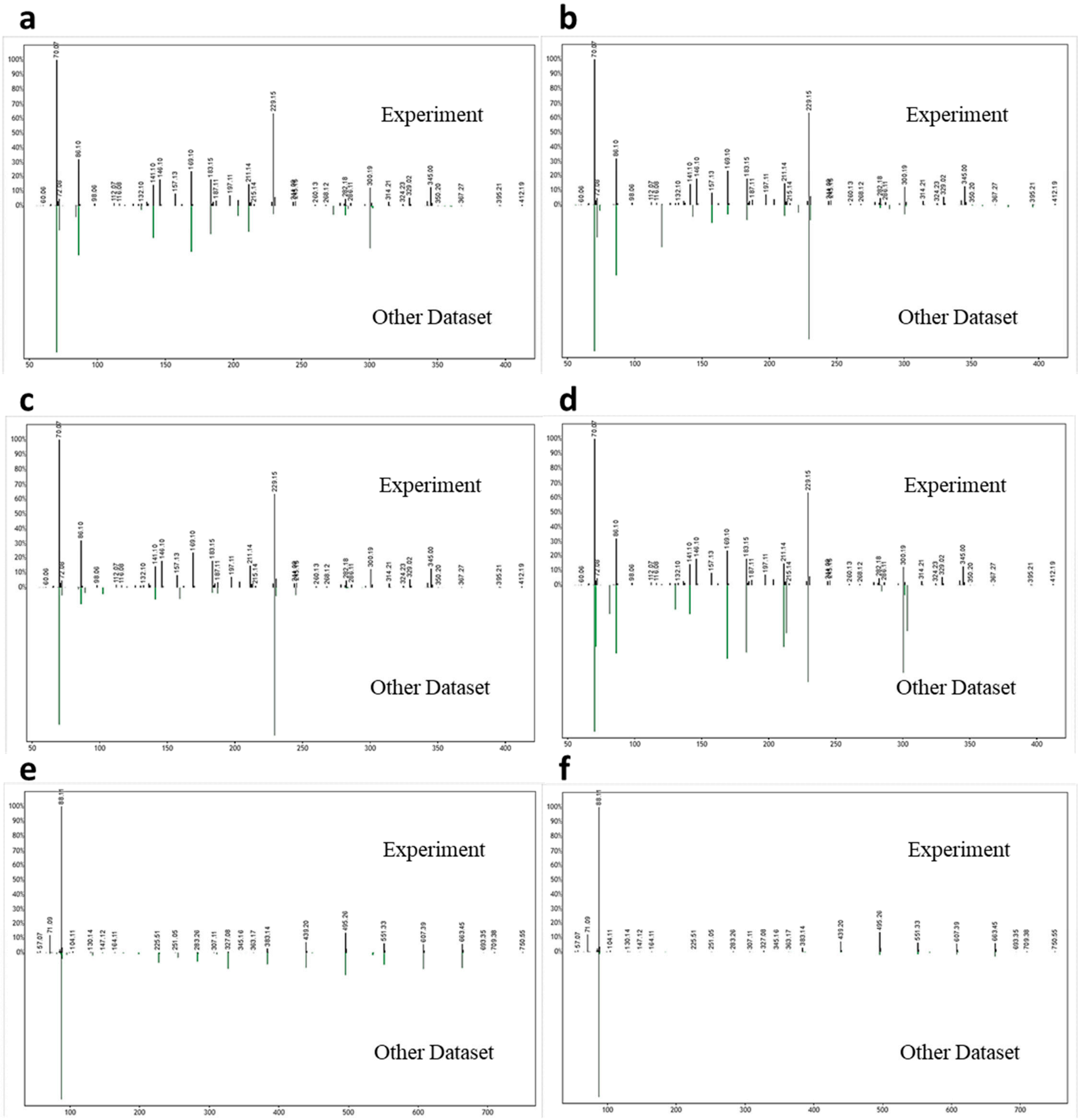

Figure S9. Comparison of the MS/MS spectra of the uncharacterized fungal metabolites in the aqueous extracts with those in the public metabolomics datasets extracted by MASST. Metabolites of sub-figures refer to **Table S8**. (continuing)

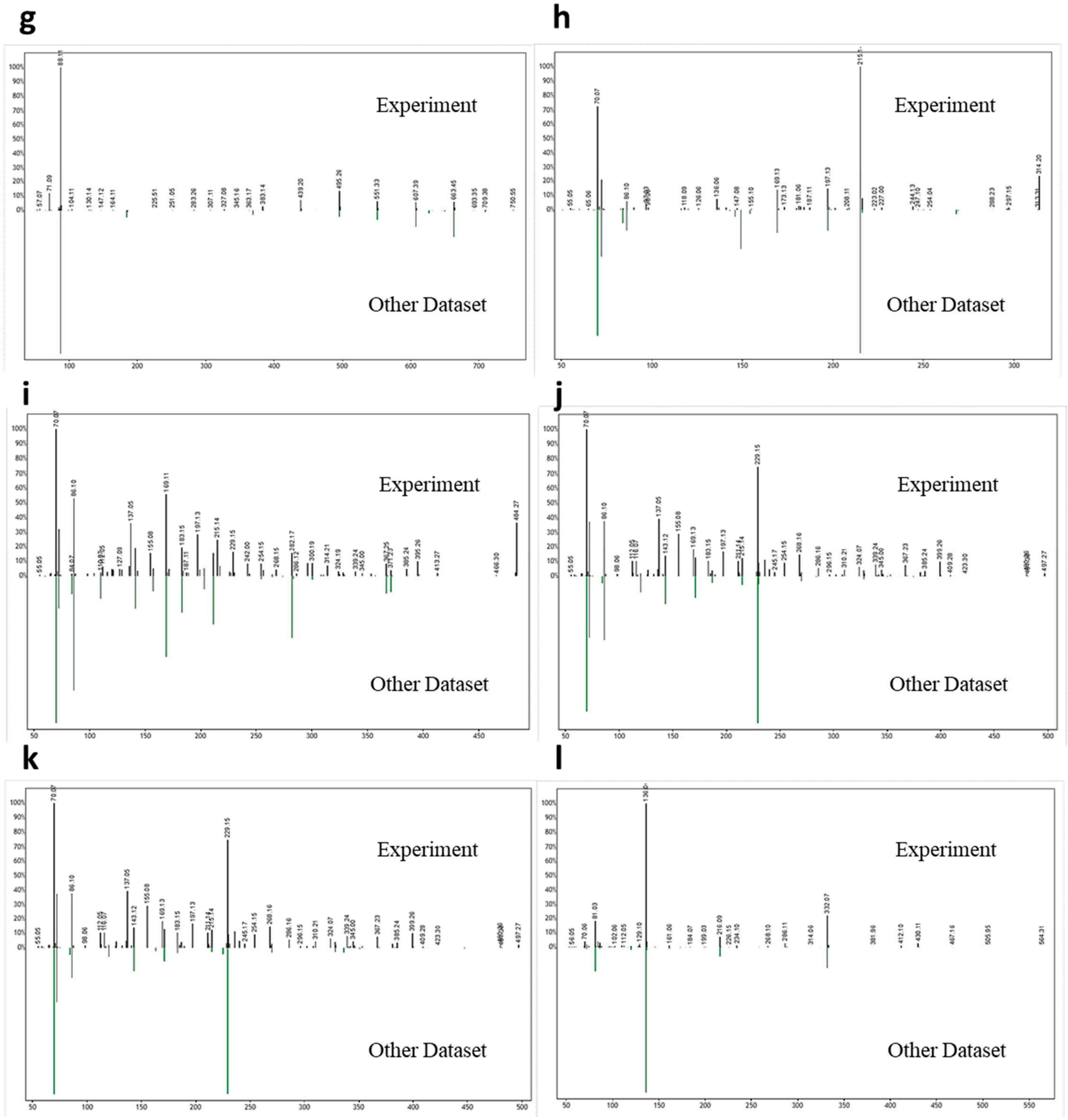

Figure S9. Comparison of the MS/MS spectra of the uncharacterized fungal metabolites in the aqueous extracts with those in the public metabolomics datasets extracted by MASST. Metabolites of sub-figures refer to **Table S8**. (continuing)

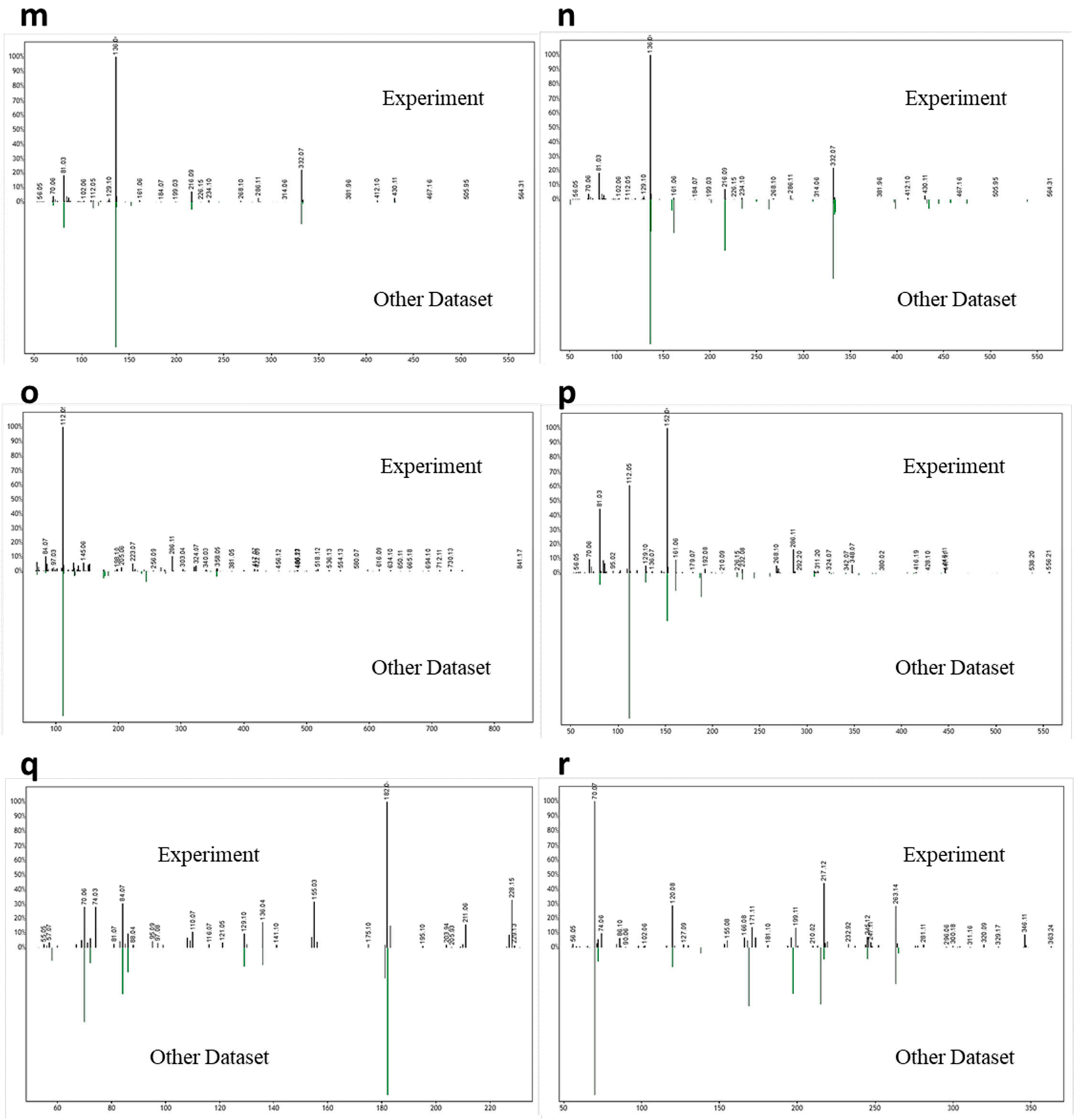

Figure S9. Comparison of the MS/MS spectra of the uncharacterized fungal metabolites in the aqueous extracts with those in the public metabolomics datasets extracted by MASST. Metabolites of sub-figures refer to **Table S8**. (continuing)

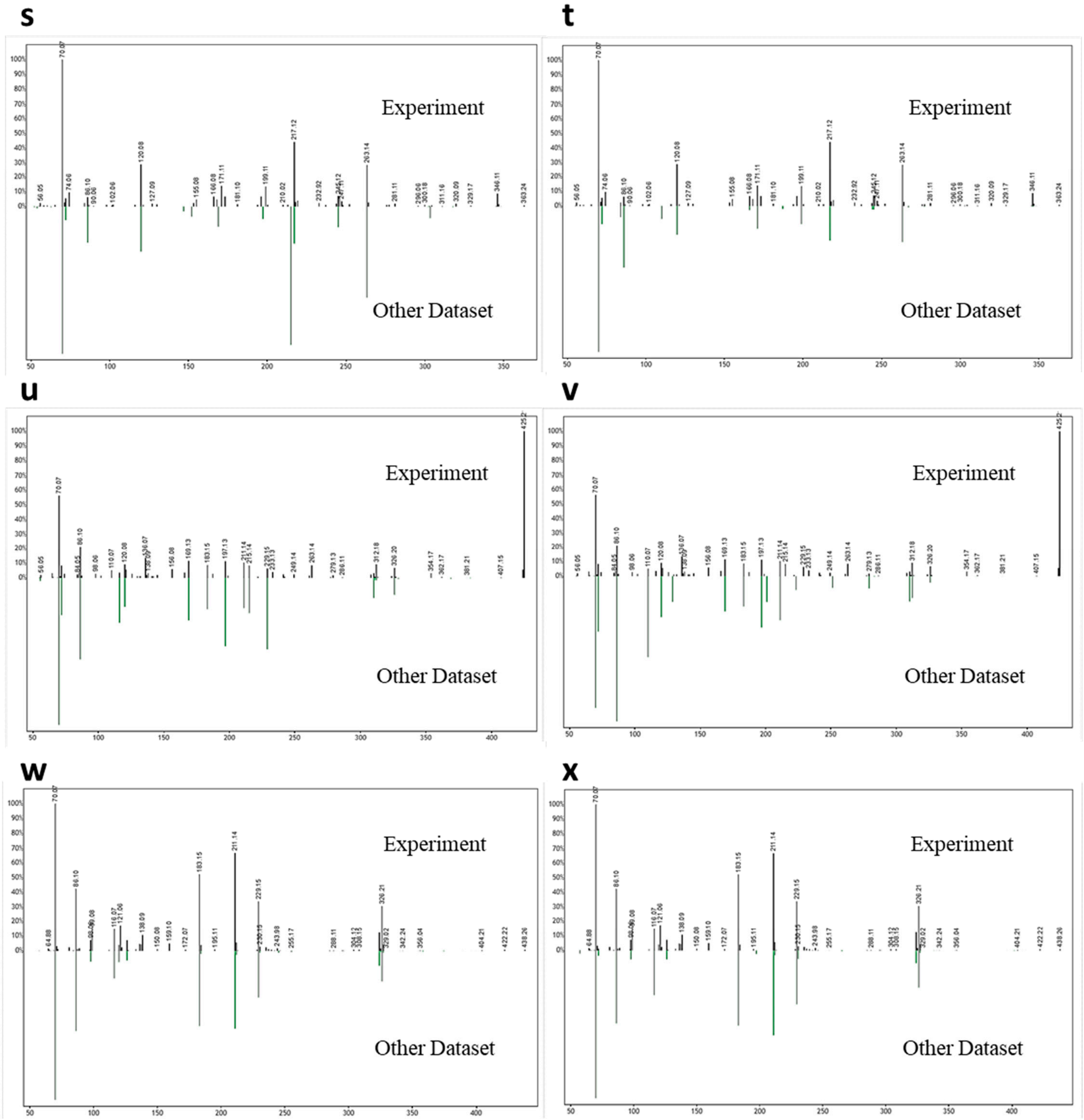

Figure S9. Comparison of the MS/MS spectra of the uncharacterized fungal metabolites in the aqueous extracts with those in the public metabolomics datasets extracted by MASST. Metabolites of sub-figures refer to **Table S8**. (continuing)

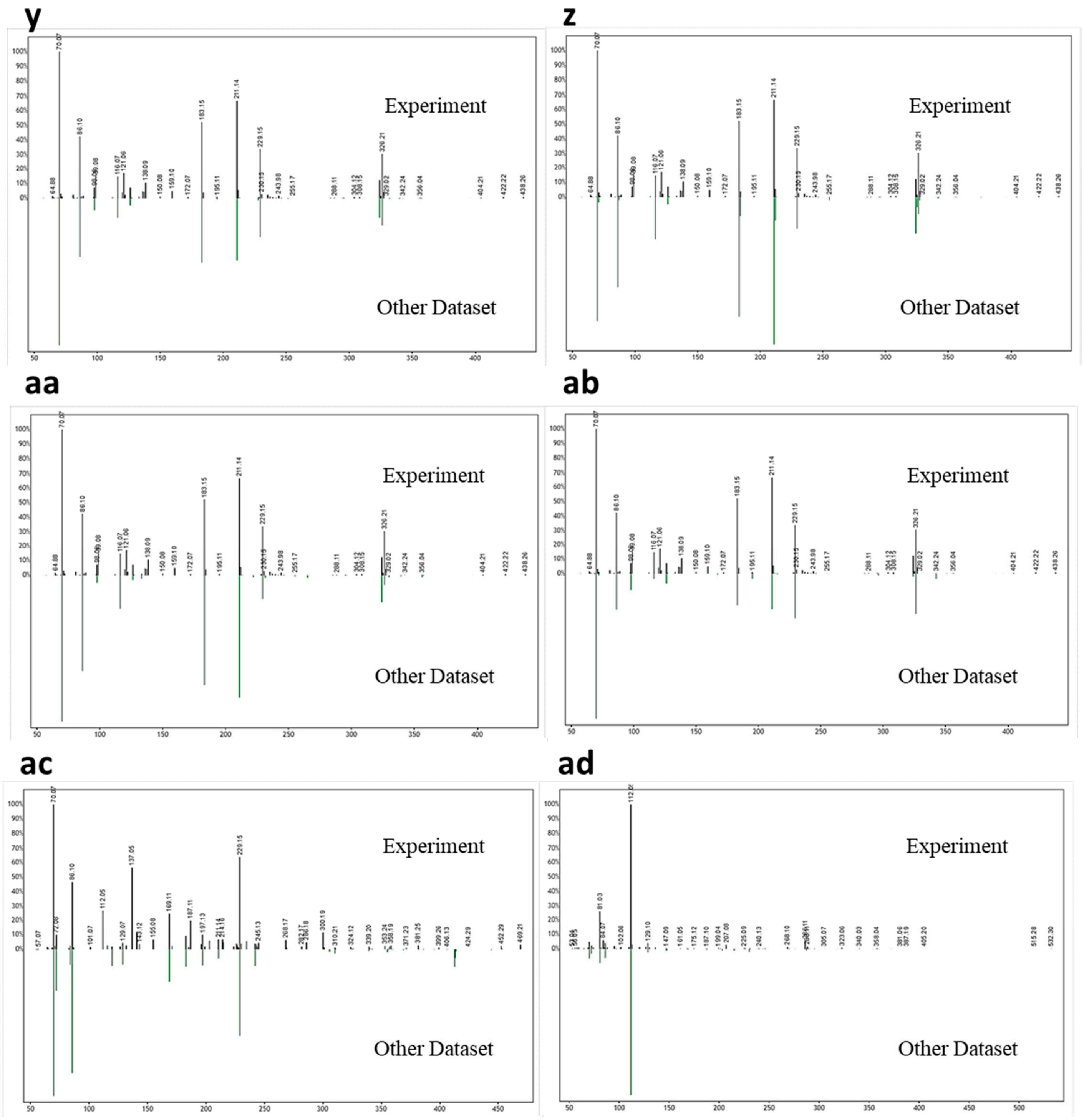

Figure S9. Comparison of the MS/MS spectra of the uncharacterized fungal metabolites in the aqueous extracts with those in the public metabolomics datasets extracted by MASST. Metabolites of sub-figures refer to **Table S8**. (continuing)

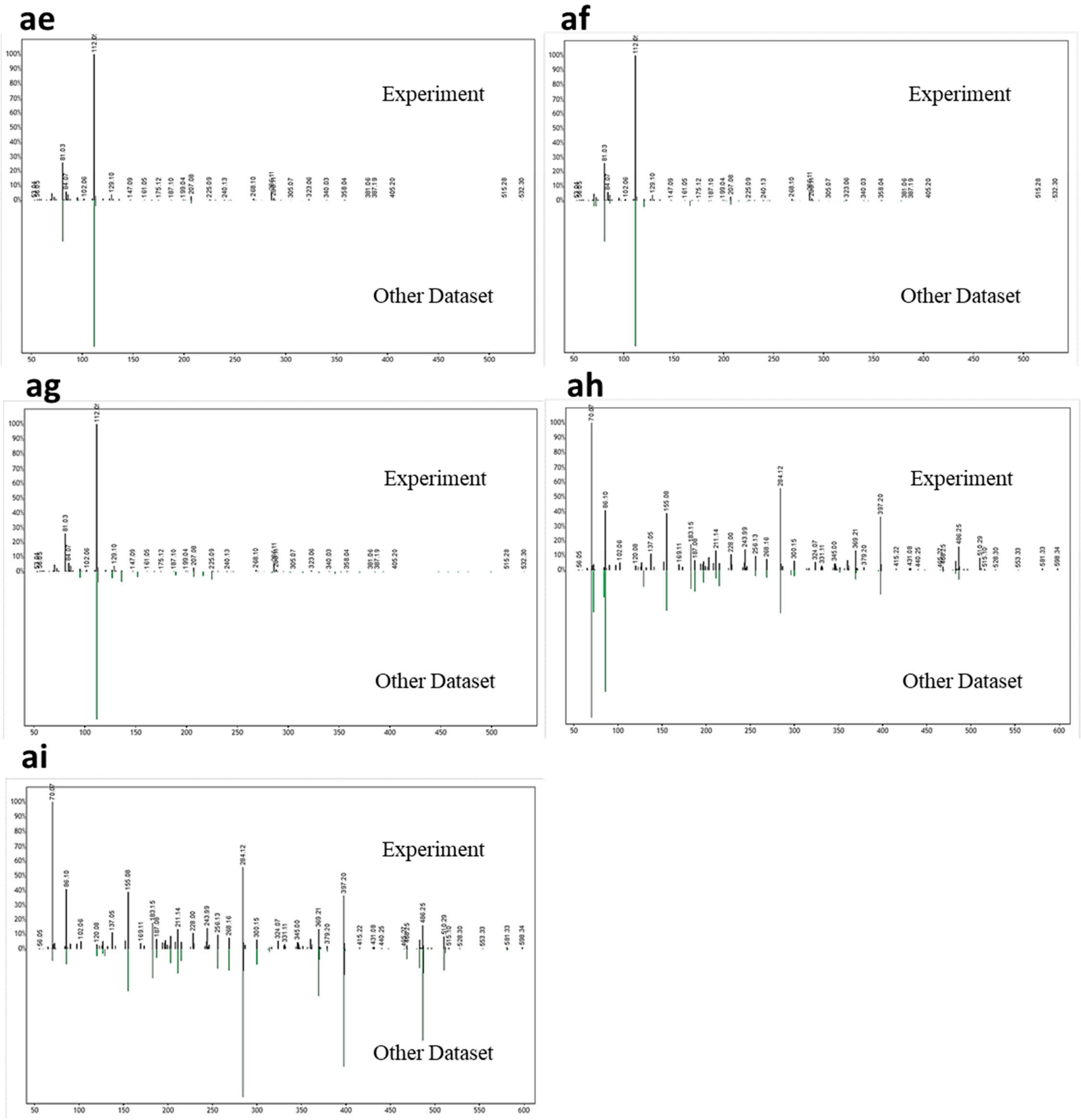

Figure S9. Comparison of the MS/MS spectra of the uncharacterized fungal metabolites in the aqueous extracts with those in the public metabolomics datasets extracted by MASST. Metabolites of sub-figures refer to **Table S8**.

(iii) References

1. Sumner, L.W.; Amberg, A.; Barrett, D.; Beale, M.H.; Beger, R.; Daykin, C.A.; Fan, T.W.M.; Fiehn, O.; Goodacre, R.; Griffin, J.L., et al. Proposed minimum reporting standards for chemical analysis. *Metabolomics* **2007**, *3*, 211-221, doi:10.1007/s11306-007-0082-2.
2. Dührkop, K.; Fleischauer, M.; Ludwig, M.; Aksenov, A.A.; Melnik, A.V.; Meusel, M.; Dorrestein, P.C.; Rousu, J.; Böcker, S. SIRIUS 4: A rapid tool for turning tandem mass spectra into metabolite structure information. *Nat. Methods* **2019**, *16*, 299-302, doi:10.1038/s41592-019-0344-8.
3. Wang, M.; Jarmusch, A.K.; Vargas, F.; Aksenov, A.A.; Gauglitz, J.M.; Weldon, K.; Petras, D.; da Silva, R.; Quinn, R.; Melnik, A.V., et al. Mass spectrometry searches using MASST. *Nat. Biotechnol.* **2020**, *38*, 23-26, doi:10.1038/s41587-019-0375-9.
